# Identification and validation of the nicotine metabolism-related signature of bladder cancer by bioinformatics and machine learning

**DOI:** 10.3389/fimmu.2024.1465638

**Published:** 2024-12-17

**Authors:** Yating Zhan, Min Weng, Yangyang Guo, Dingfeng Lv, Feng Zhao, Zejun Yan, Junhui Jiang, Yanyi Xiao, Lili Yao

**Affiliations:** ^1^ Department of Blood Transfusion, The First Affiliated Hospital of Ningbo University, Ningbo, China; ^2^ Department of Urology, The First Affiliated Hospital of Ningbo University, Ningbo, China; ^3^ Department of Thyroid and Breast Surgery, The First Affiliated Hospital of Ningbo University, Ningbo, China; ^4^ Department of Thyroid and Breast Surgery, The Second Affiliated Hospital of Shanghai University, Wenzhou, China; ^5^ Department of Ultrasonography, The First Affiliated Hospital of Wenzhou Medical University, Wenzhou, China

**Keywords:** nicotine metabolism, bladder cancer, machine learning, prognostic signature, immunotherapy benefit

## Abstract

**Background:**

Several studies indicate that smoking is one of the major risk factors for bladder cancer. Nicotine and its metabolites, the main components of tobacco, have been found to be strongly linked to the occurrence and progression of bladder cancer. However, the function of nicotine metabolism-related genes (NRGs) in bladder urothelial carcinoma (BLCA) are still unclear.

**Methods:**

NRGs were collected from MSigDB to identify the clusters associated with nicotine metabolism. Prognostic differentially expressed genes (DEGs) were filtered via differentially expression analysis and univariate Cox regression analysis. Integrative machine learning combination based on 10 machine learning algorithms was used for the construction of robust signature. Subsequently, the clinical application of signature in terms of prognosis, tumor microenvironment (TME) as well as immunotherapy was comprehensively evaluated. Finally, the biology function of the signature gene was further verified via CCK-8, transwell migration and colony formation.

**Results:**

Three clusters associated with nicotine metabolism were discovered with distinct prognosis and immunological patterns. A four gene-signature was developed by random survival forest (RSF) method with highest average Harrell’s concordance index (C-index) of 0.763. The signature exhibited a reliable and accurate performance in prognostic prediction across TCGA-train, TCGA-test and GSE32894 cohorts. Furthermore, the signature showed highly correlation with clinical characteristics, TME and immunotherapy responses. Suppression of MKRN1 was found to reduce the migration and proliferation of bladder cancer cell. In addition, enhanced migration and proliferation caused by nicotine was blocked down by loss of MKRN1.

**Conclusions:**

The novel nicotine metabolism-related signature may provide valuable insights into clinical prognosis and potential benefits of immunotherapy in bladder cancer patients.

## Introduction

Globally, bladder cancer is one of the ten most prevalent types of carcinomas and poses a severe threat to health (1). Bladder cancer can be categorized into two main types based on the extent of invasion: muscle-invasive bladder cancer (MIBC) and non-muscle-invasive bladder cancer (NMIBC). NMIBC is the most common type of bladder cancer, accounting for approximately 75% of bladder cancer. NMIBC is associated with a high risk of recurrence and disease progression, with reported 5-year recurrence rates of 50-70% and 10-30% of patients experiencing disease progression within 5 years (2). It has been reported that 10-15% of NMIBC may develop to MIBC, accompanying with an elevated risk of metastasis and a relatively lower 5-year survival rate (3). It has been proven that numerous biomarkers and scoring systems have been used to predict the prognosis or progression of bladder cancer patients (4, 5). The treatments for NMIBC patients include transurethral resection of bladder tumor, intravesical chemotherapy, intravesical immunotherapy and follow-up and surveillance of NMIBC patients (6). On the other hand, MIBC is treated with radical cystectomy, chemotherapy and radiotherapy (7). Recently, immunotherapy has been gradually applied in bladder cancer. However, the highly heterogeneous of pathogenesis and clinical manifestation pose a threat to curative effect of immunotherapy in bladder urothelial carcinoma (BLCA) (8, 9). Therefore, it is critical to identify effective diagnostic and prognostic biomarkers for predicting the sensitivity of immunotherapy.

Tobacco smoking is recognized as a significant contributing factor to the development of bladder cancer, with an estimated involvement in around 50-65% of annual new diagnoses (10). Research has demonstrated that smoking significantly elevates the likelihood of developing bladder cancer by a factor of three to four. Nicotine, an addictive substance of tobacco, exists at high concentrations in the bloodstream and urine of smokers (11). Nicotine is metabolized in the liver, primarily by the liver cytochrome P450 enzymes CYP2A6 and CYP2B6 to cotinine (12). Moreover, nicotine and its metabolites including cotinine show strongly tumor-promoting effects, such as cell proliferation, angiogenic growth and cell survival. Continued smoking is highly associated with a higher incidence and contributes to tumor recurrence, progression, and acquired chemotherapy resistance in bladder cancer. Yuge et al. discovered that nicotine promotes tumor growth and leads to acquired chemoresistance through the activation of PI3K/Akt/mTOR pathway in bladder cancer (13). Although smoking and nicotine are well-established triggers for bladder cancer, the specific mechanisms by which nicotine and nicotine metabolism-related genes (NRGs) induce bladder cancer, promote recurrence and lead to treatment resistance are still unclear. To better evaluate prognosis and provide individualized therapy for patients with BLCA, a nicotine metabolism related signature (NRS) was urgently developed.

In this study, three nicotine metabolism related-clusters were identified with different prognosis and tumor immune microenvironment (TME). A robust NRS with four NRGs was developed based on machine learning combination, which was highly correlated with clinical characteristics, TME and immunotherapy responses. Our study may aid in the recognition of high-NRS patients, guide the implementation of immunotherapy, and further improve the survival outcomes for individuals with BLCA.

## Results

### Identification of three nicotine metabolism-related clusters

**Figure 1:**
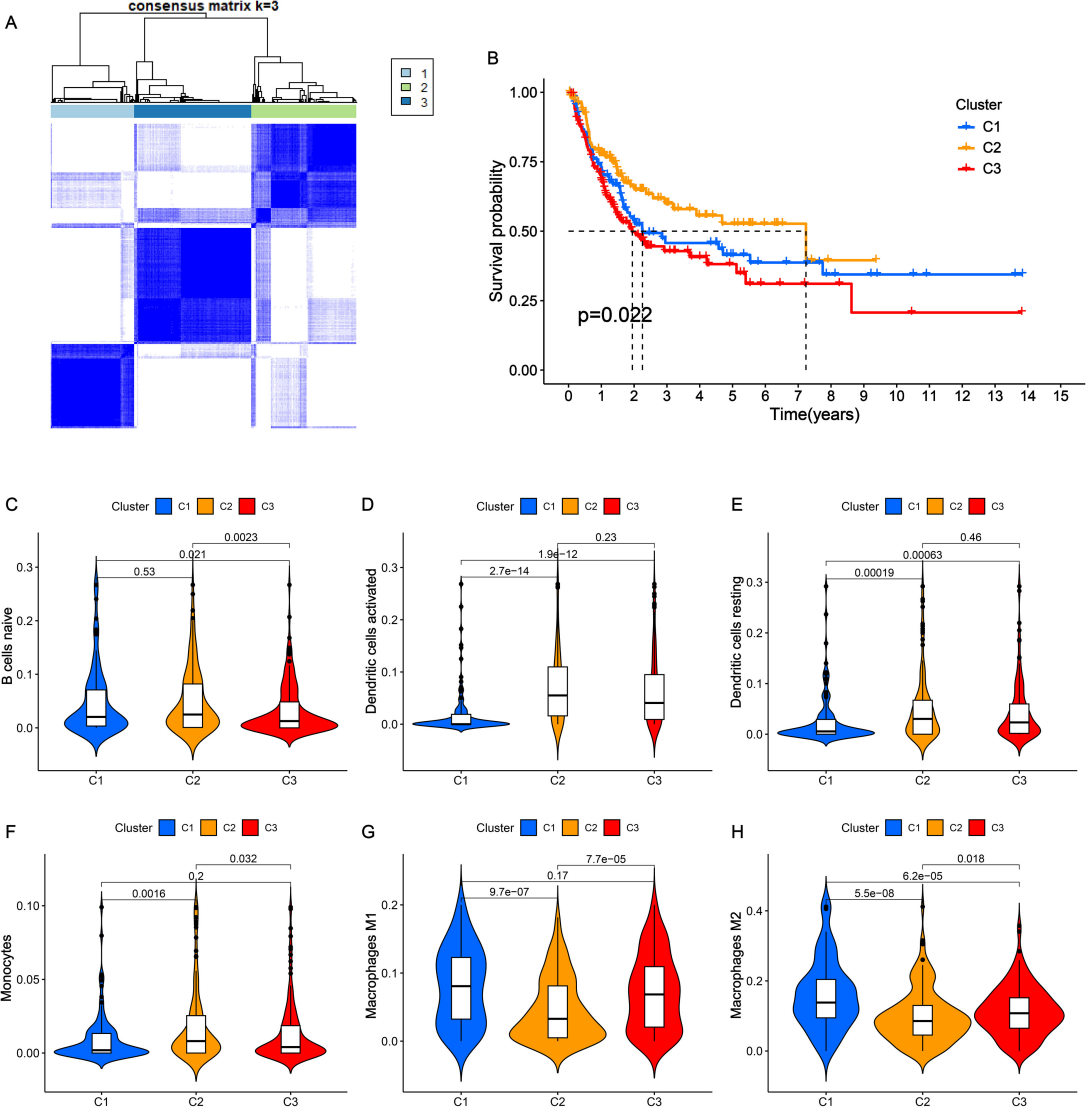
Consensus clustering of NRGs in BLCA. **(A)** The clustering diagram for defining the three clusters. **(B)** K-M survival curve of the three clusters. **(C-H)** The infiltration levels of naïve B cells **(C)**, activated dendritic cells **(D)**, resting dendritic cells **(E)**, monocytes **(F)**, macrophages M1 **(G)** and macrophages M2 **(H)** among three clusters.

To explore the impact of NRGs on the classification of BLCA patients, unsupervised clustering analysis was performed based on the expression of 63 NRGs. Significantly, BLCA patients were classified into three nicotine metabolism related-clusters in the Cancer Genome Atlas (TCGA), which maintained a relatively stable modification pattern (**Figure 1A**). To disclose the correlation between clusters and prognosis, Kaplan-Meier (K-M) survival curve of overall survival (OS) was utilized to compare the difference across three clusters. The results of K-M curve revealed an obvious difference among three clusters, which cluster 2 exhibiting the highest OS rate and cluster 3 displaying the lowest OS rate (**Figure 1B**). Additionally, the immune cell infiltration status of three cluster was investigated via CIBERSORT algorithm (**Figures 1C–H**). Cluster 1 had high infiltration of M1 and M2 macrophage. Cluster 2 showed the highest numbers of B cell, activated dendritic cell, resting dendritic cell, monocytes. These results reveal that the identified nicotine metabolism related-clusters are highly associated with prognosis and TME.

### Integrative machine learning algorithms developed an optimal NRS

To further investigate the gene expression profile in the three distinct nicotine metabolism related-clusters modification patterns, differential expression analysis was performed among the three clusters. Then, all differentially expressed genes (DEGs) were intersected and 651 DEGs were found after intersection. Afterwards, a total of 331 genes were identified, exhibiting significant variations in expression levels between BLCA and normal tissues. In order to facilitate further analysis and prediction, we randomly divided the TCGA-BLCA cohort into two cohorts at a 1:1 ratio: TCGA-train and TCGA-test cohorts. In TCGA-train cohort, 91 prognostic DEGs were selected from 331 genes via univariate Cox regression analysis. The 91 prognostic DEGs were then submitted to the integrative machine learning procedure to identify the optimal NRS with the highest sensitivity and accuracy. As shown in **Supplementary Figure S1A**, a total of 100 kinds of prognostic signatures were acquired in TCGA-train cohort, and we further assessed the Harrell’s concordance index (C-index) of each signature across TCGA-test and GSE32894 cohorts. Obviously, the NRS developed by random survival forest (RSF) showed a highest average C-index of 0.763. Simultaneously, the optimal NRS identified by RSF contained 4 mRNAs, including AHNAK, MKRN1, SFXN4 and ZMYND8 (**Supplementary Figure S1B**).

### Evaluation of the prognostic value of NRS

**Figure 2:**
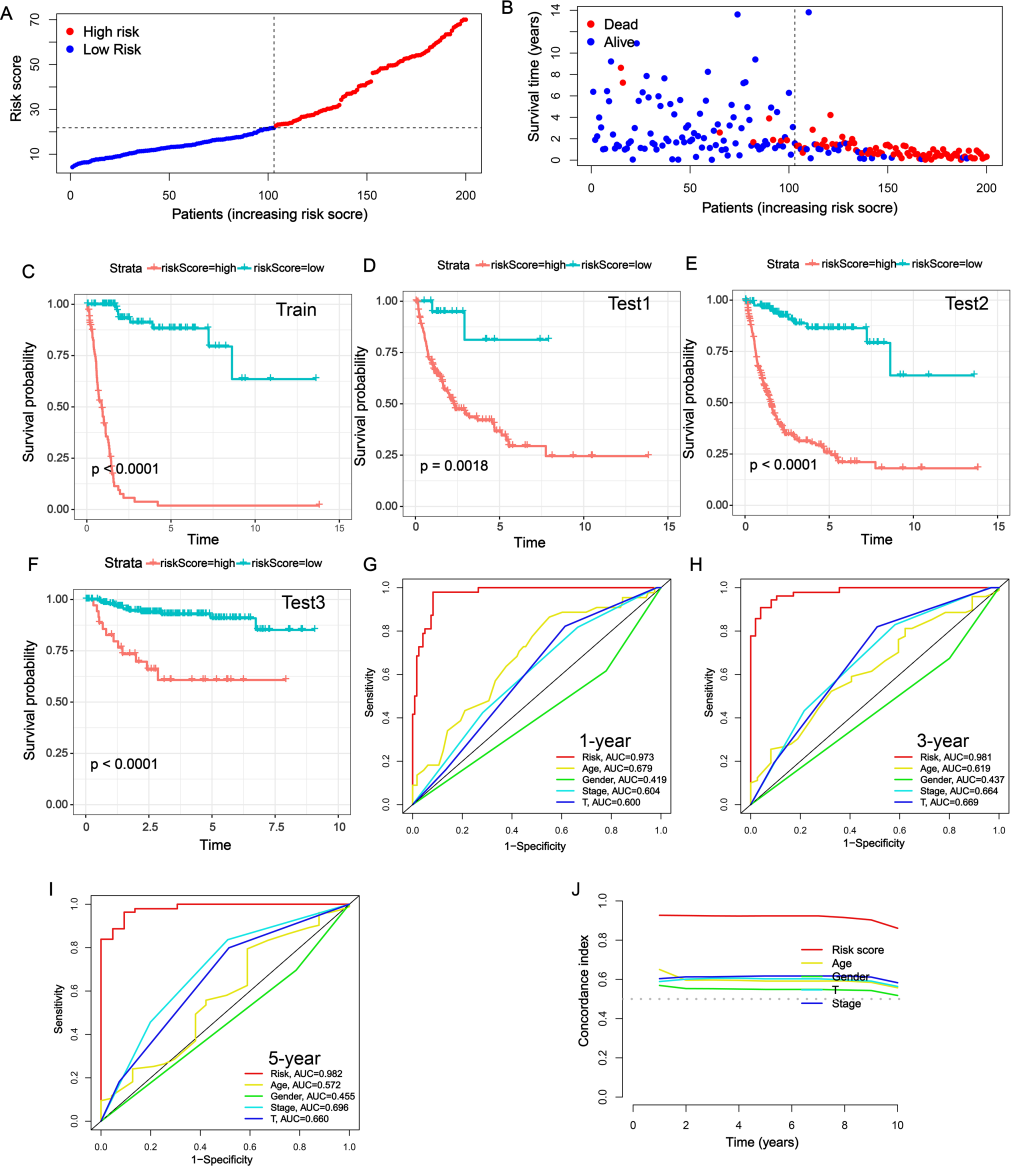
Evaluation of the prognostic value of NRS. **(A)** NRS scores of BLCA patients across TCGA-train cohort. **(B)** Survival status of BLCA patients across TCGA-train cohort. **(C-F)** K-M survival curves of BLCA patients across TCGA-train **(C)**, TCGA-all **(D)**, TCGA-test **(E)** and GSE32894 **(F)** cohorts. **(G-I)** Multi-index ROC curves of NRS and other clinical characteristics in 1-, 3- and 5-year. **(J)** The C-index of NRS and other clinical characteristics in predicting the prognosis of BLCA patients in TCGA-train cohort.

Next, we further evaluated the prognostic value of the NRS constructed by RSF. Each BLCA patient received an NRS score via NRS and was then divided into high- and low-NRS groups based on the best cut-off value (**Figure 2A**). Low NRS score patients exhibited longer OS than patients with high NRS in TCGA-train cohort (**Figure 2B**). Moreover, the results of K-M survival curve showed BLCA patients with high NRS displayed lower survival rates in TCGA-train cohort (**Figure 2C**). Similarly, survival analysis in the TCGA-all, TCGA-test, and GSE32894 datasets confirmed the robustness of the signature (**Figures 2D–F**). Moreover, we further validated the prognostic value of NRS in GSE13507 and GSE31684 datasets, and similar results were observed (**Supplementary Figure S2**). To further evaluate the superiority of the NRS than other clinical characteristics, receiver operating characteristic (ROC) curves were performed. The area under the curve (AUC) value of NRS in 1-, 3-, and 5-year was 0.973, 0.981 and 0.982, higher than other clinical characteristics (**Figures 2G–I**). In addition, C-index line chart demonstrated that the NRS had a higher C-index value compared to other clinical characteristics, indicating its strong prognostic value (**Figure 2J**). It has been reported that a number of prognostic signatures have been developed to predict the prognosis of BLCA patients. We collected 6 published prognostic signatures for BLCA and calculated the corresponding AUC and C-index values. Interestingly, the AUC and C-index values of our NRS demonstrated a significant superiority compared to other published signatures (**Supplementary Figure S3**). Overall, these data indicate that our NRS may be a potential prognostic predictor in BLCA.

### Clinical correlation analysis and stratified survival analysis

To analyze the association between NRS and clinical characteristics, the distribution of NRS scores across different clinical characteristics subgroups was assessed. It was found that the change of NRS was highly correlated with age, gender, N stage, tumor stage and T stage (**Supplementary Figure S4**). In addition, the stratified survival analysis was further carried out to investigate the clinical applicability of NRS. The results of **Supplementary Figure S5** demonstrated that low-NRS patients possessed a better survival probability than those with high NRS in clinical subgroups of age > 65 years, age ≤65 years, female, male, M0 stage, N0 stage, N1-3 stage, tumor stage I–II, tumor stage III–IV, T1-2 stage and T3-4 stage.

### Construction of nomogram

**Figure 3:**
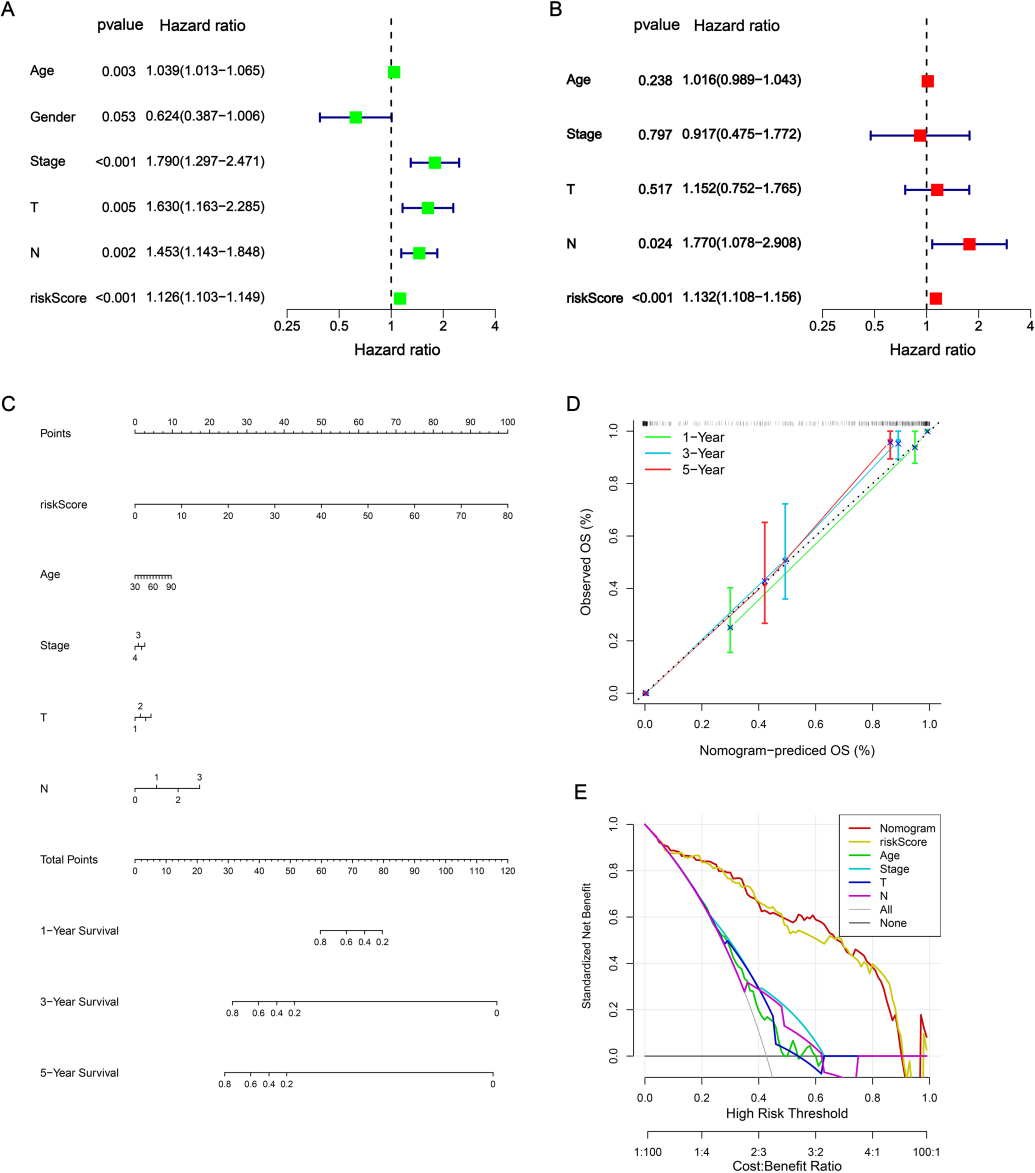
Construction of nomogram. **(A, B)** Independent prognostic analysis via univariate Cox analysis and multivariate Cox regression analysis. **(C)** Nomogram. **(D)** Calibrations curves for predicting 1-, 3-, and 5-year OS. **(E)** DCA curves.

In the univariate Cox analysis, the NRS and clinical characteristics (age, tumor stage, T stage and N stage) were considered as risk factors for BLCA patients in TCGA-train cohort (**Figure 3A**). In addition, the results of multivariate Cox regression analysis demonstrated that NRS was proven as an independent prognostic factor (**Figure 3B**). Subsequently, nomogram was developed based on risk factors (NRS, age, tumor stage, T stage and N stage) for improvement of the predictive performance in a patient’s prognosis (**Figure 3C**). Each BLCA patient can obtain a total score for the prediction of 1-, 3- and 5-year OS. The calibration plots indicated that this nomogram exhibited a robust and independent predictive probability, which was closed to the actual OS (**Figure 3D**). The decision curve analysis (DCA) further demonstrated that nomogram showed an efficient net benefit in predicting OS, implying its potential to improve OS prediction over traditional prognostic markers (**Figure 3E**).

### Evaluation of TME

**Figure 4:**
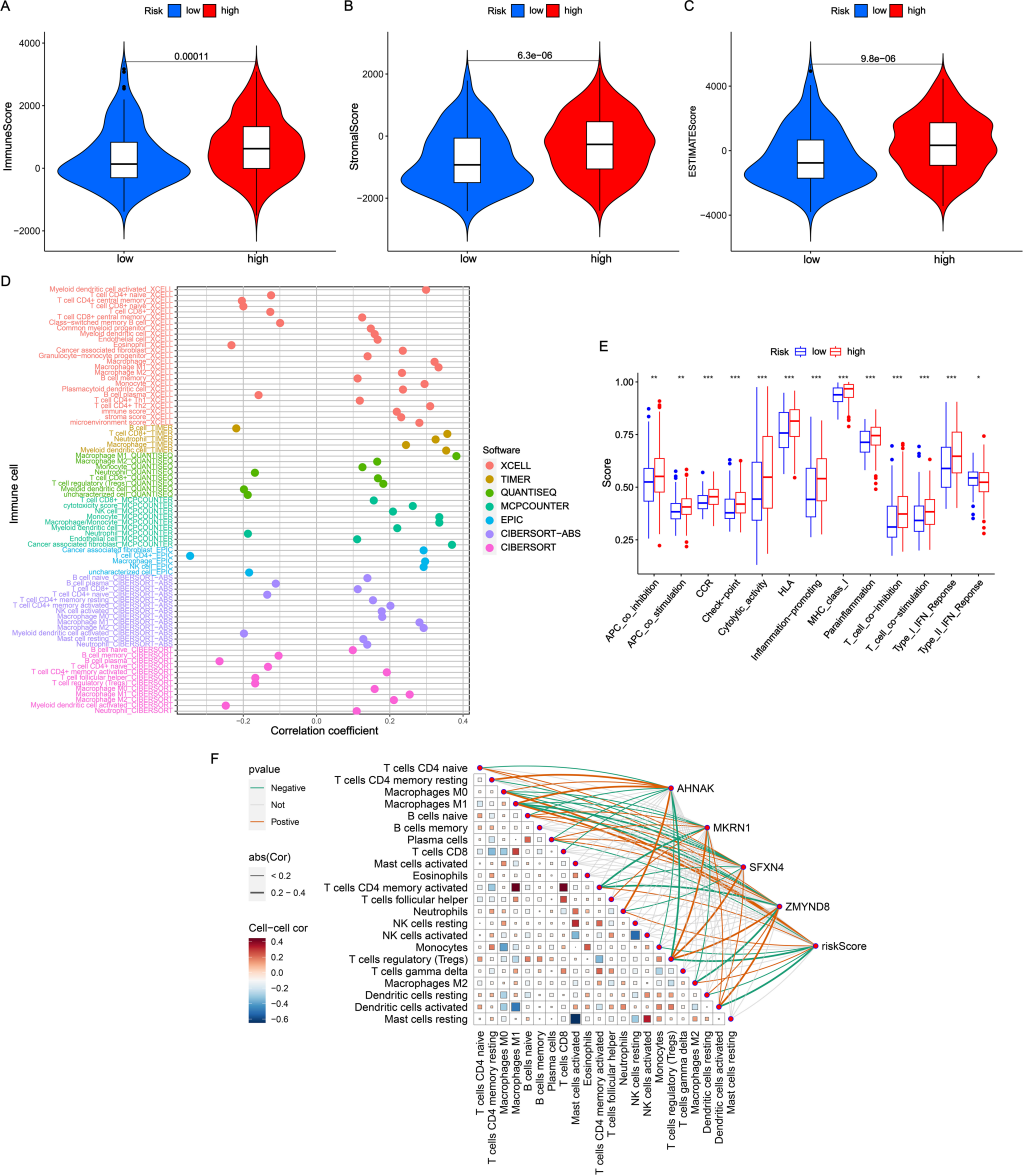
Evaluation of TME in NRS. **(A-C)** Comparisons of immune score, stromal score and estimate score between high- and low-NRS groups. **(D)** Correlation analysis of NRS scores with diverse immune cells. **(E)** Distribution of immune function scores varied NRS subgroups. **(F)** Correlation analysis of NRS scores and four signature genes expressions with immune cells. *P<0.05; **P<0.01; ***P<0.001.

TME, consisting immune and stromal cells, is highly involved in the progression of BLCA (14). As shown in **Figures 4A-C**, high-NRS patients had higher immune score, stromal score and estimate score compared with low-NRS group. Next, a bubble chart revealed that NRS processed high correlation with the abundance of immune cells via 7 immune algorithms (**Figure 4D**). It was obvious that NRS was positively associated with the level of cancer associated fibroblast (CAF), macrophage, monocyte, plasmacytoid dendritic cell, NK cell, Th2 cell, immune score, stroma score, microenvironment score and cytotoxicity score (coefficient > 0.2). Meanwhile, NRS showed a negative correlation with central memory CD4^+^ T cell, eosinophil and plasma B cell (coefficient < −0.2). Moreover, the distribution of immune function scores between high- and low-NRS groups was conducted. **Figure 4E** indicated that the high-NRS group displayed elevated levels of immune functions, except for type II IFN response. Based on CIBERSORT algorithm, the correlation among the infiltration levels of 22 immune cell types were calculated (**Figure 4F**). Meanwhile, we found that the coefficients of NRS and signature genes with almost immune cells were significant.

### Immunotherapy response

**Figure 5:**
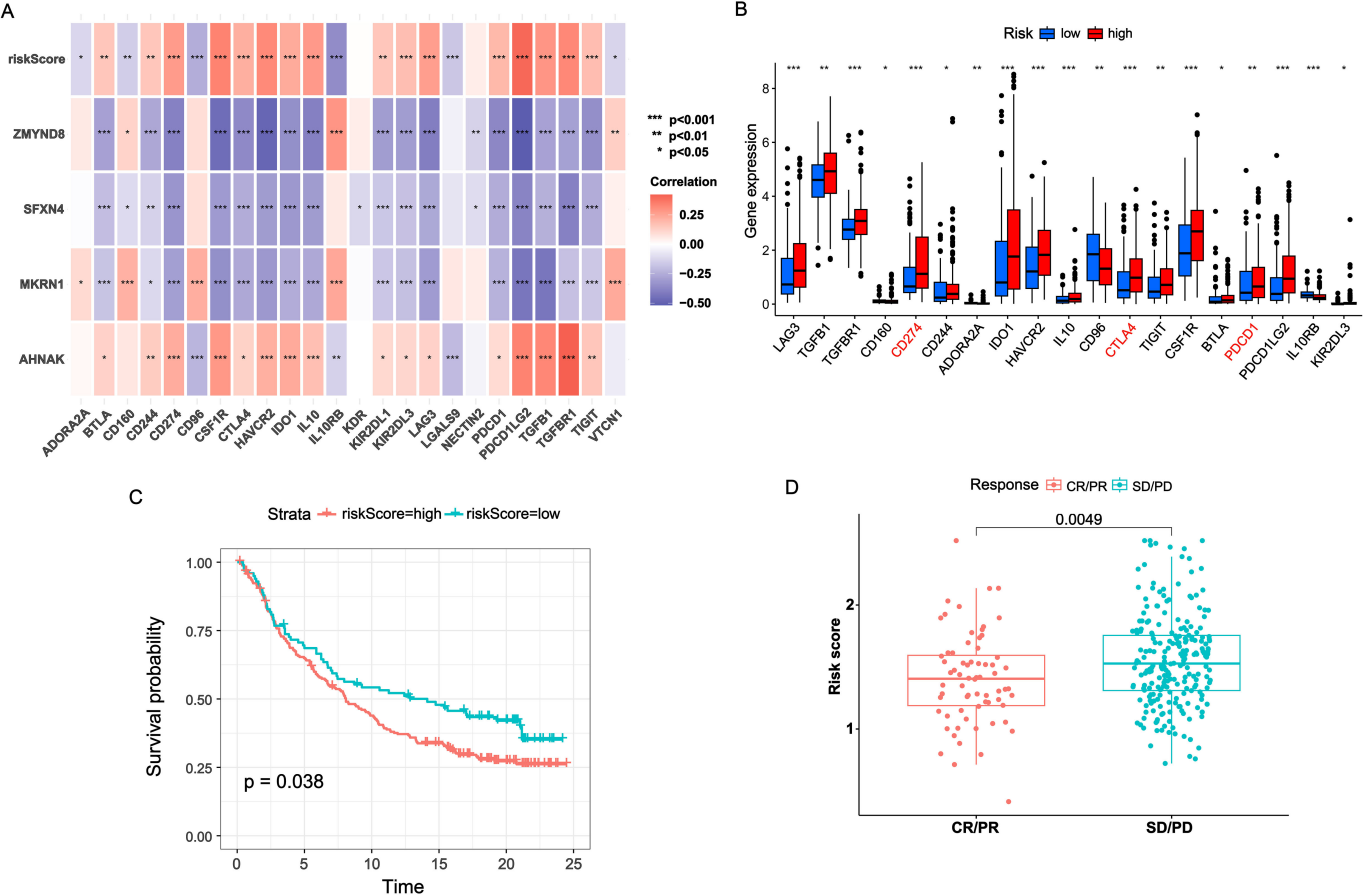
Immunotherapy benefits of NRS. **(A)** Correlation heatmap of NRS scores and four signature genes expressions with immune checkpoints expressions. **(B)** Boxplot of immune checkpoints expression across varied NRS groups. **(C)** K-M survival analysis across varied NRS groups in IMvigor210 cohort. **(D)** Distribution of NRS scores between CR/PR and SD/PD. *P<0.05; **P<0.01; ***P<0.001.

Recently, immunotherapy has been proven effective in the treatment of virous solid tumors (15). Considering the roles of immune checkpoints in immunotherapy, we investigated the association between NRS and immune checkpoint genes. As shown in **Figure 5A**, there was positive correlation of NRS and AHNAK with majority immune checkpoints. By contrast, MKRN1, SFXN4 and ZMYND8 had remarkably negative correlation with almost immune checkpoints. The majority of immune checkpoints, especially PD-1, PD-L1 and CTLA4, exhibited increased expression levels in the high-NRS group compared to the low-NRS group (**Figure 5B**). Additionally, the predictive value of NRS in immunotherapy was validated in the IMvigor210 cohort. In the IMvigor210 cohort, the low-NRS group had a good performance in OS than high-NRS group (**Figure 5C**). Moreover, we found that the NRS score in non-responders (stable disease or progressive disease [SD/PD]) was remarkably higher versus responders (complete response or partial response [CR/PR]) (**Figure 5D**).

### Function enrichment analysis

**Figure 6:**
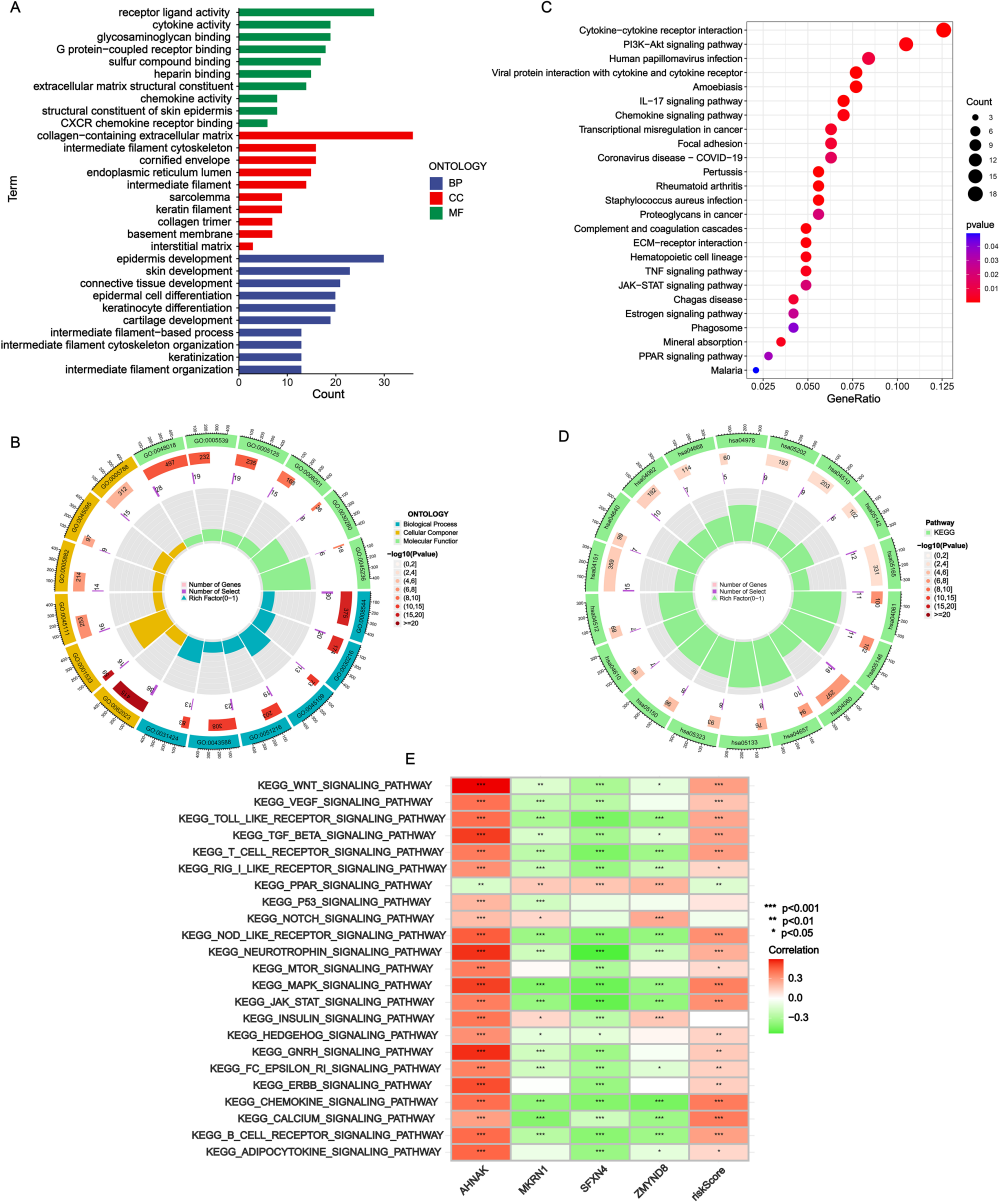
Functional enrichment analysis. **(A, B)** GO analysis. **(C, D)** KEGG analysis. **(E)** Correlation heatmap of NRS scores and four signature genes expressions with GSVA scores of KEGG pathways. *P<0.05; **P<0.01; ***P<0.001.

Gene Ontology (GO), Kyoto Encyclopedia of Genes and Genomes (KEGG) and gene set variation analyses (GSVA) analyses were conducted to further disclose the NRS-related potential molecular mechanisms. In the results of GO enrichment, the DEGs between the two subgroups were mainly associated with the interaction of various molecule (such as receptor/ligand activity, cytokine activity, chemokine activity, glycosaminoglycan binding and G protein-coupled receptor binding) and the development of tissue cell (epidermis development, skin development, epidermal cell differentiation and keratinocyte differentiation) (**Figures 6A, B**). KEGG revealed significant enrichment of cytokine/cytokine receptor interaction, chemokine signaling pathway, PI3k-Akt signaling pathway and IL-17 signaling pathway (**Figures 6C, D**). In addition, the correlation of KEGG pathways with NRS scores/signature genes were explored (**Figure 6E**). MAPK signaling pathway, JAK-STAT signaling pathway, chemokine signaling pathway and calcium signaling pathway were highly associated with NRS score.

### Validation of signature genes expression patterns through scRNA-seq analysis and *in vitro* experiment

**Figure 7:**
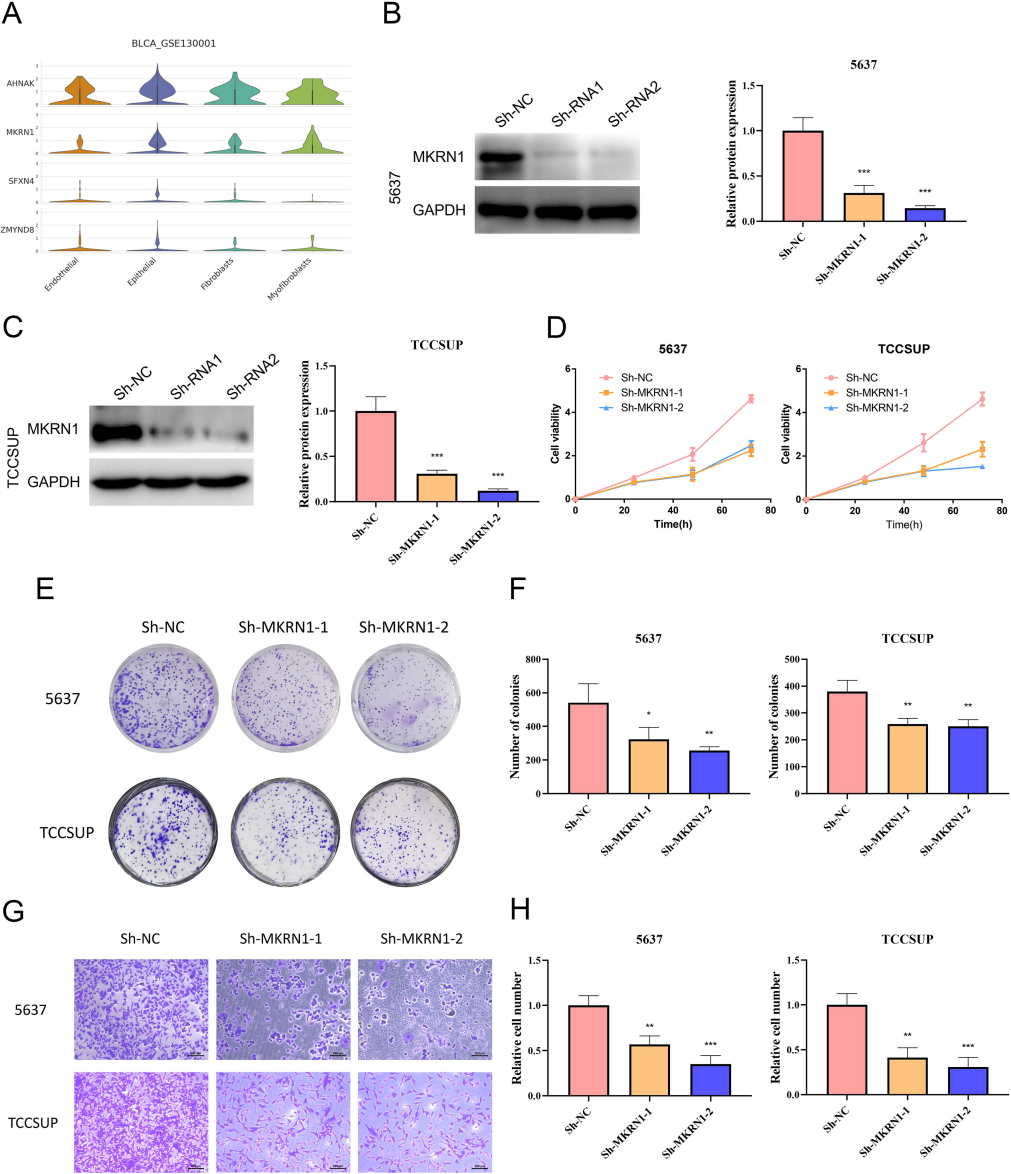
Biology role of MKRN1 in bladder cancer. **(A)** Expressions of AHNAK, MKRN1, SFXN4 and ZMYND8 in diverse cell types of single cell data (BLCA-GSE130001). **(B, C)** Validation of the efficiency of MKRN1 knock-down. **(D)** CCK-8 assay. **(E, F)** Colony formation assay. **(G, H)** Transwell migration assay. *P<0.05; **P<0.01; ***P<0.001.

**Figure 8:**
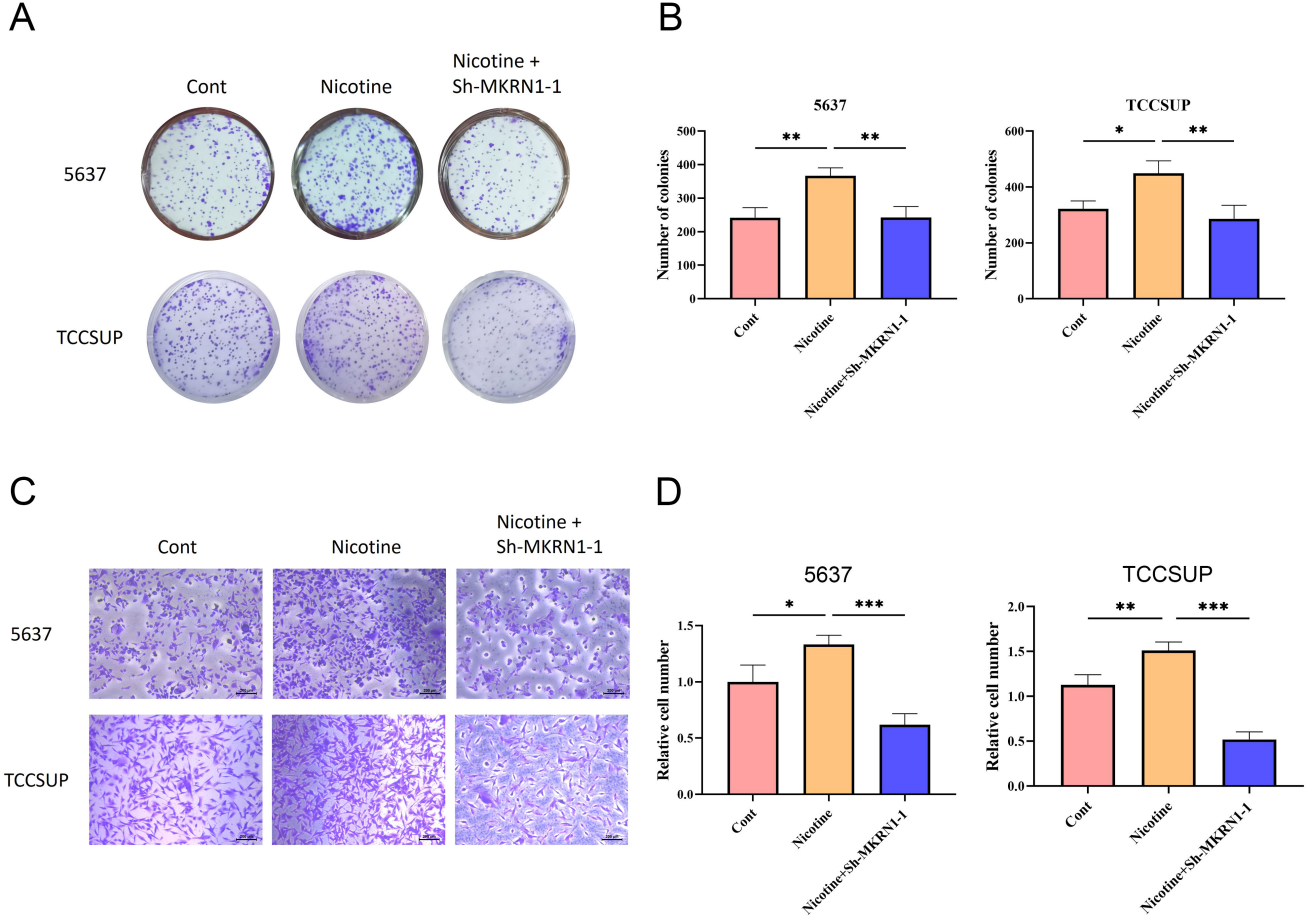
Nicotine promoted the proliferation and migration of bladder cancer cells via MKRN1. **(A, B)** Colony formation assay. **(C, D)** Transwell migration assay. *P<0.05; **P<0.01; ***P<0.001.

To further validate the particular cell types expressing the signature genes in the NRS within the TME, the scRNA-seq dataset of BLCA_GSE130001 was conducted to monitor the expression of the 4 signature genes via the Tumor Immunization Single Cell Center (TISCH) online tool (**Figure 7A**). It was found that SFXN4 and ZMYND8 displayed low expression levels in tumorigenic cells. AHNAK and MKRN1 were predominantly expressed in tumorigenic cells, especially epithelial cells. Previously, AHNAK has been recognized as a novel candidate biomarker for BLCA (16). However, the biology role of MKRN1 has not been explored in BLCA. Therefore, we selected MKRN1 for the further experimental verification. To examine whether MKRN1 participates in bladder cancer progression, bladder cancer cells were treated with sh-MKRN1 to silence MKRN1 level. Clearly, MKRN1 was significantly reduced by sh-MKRN1-1 and sh-MKRN1-2 in two human bladder cancer cell lines (5637, TCCSUP) (**Figures 7B, C**). After knocking down MKRN1, the cell proliferations of bladder cancer cells were significantly decreased (**Figure 7D**). The results of colony formation assays showed that MKRN1 knockdown reduced clone survival rate (**Figures 7E, F**). Moreover, silencing MKRN1 led to the inhibition of bladder cancer cell migration (**Figures 7G, H**). Subsequently, we further validated whether nicotine mediates the progression of bladder cancer through MKRN1. Interestingly, the proliferation and migration abilities were enhanced in nicotine-treated bladder cancer cell, while this effect was blocked down by loss of MKRN1 (**Figure 8**).

## Discussion

Tobacco smoking is an addictive behavior and epidemiological research has established a correlation between smoking and various types of cancer, including bladder cancer (17). Tobacco smoking is an important risk factor for bladder cancer. Nicotine, the complex carcinogens among tobacco, can be excreted into urine, leading to the development of bladder cancer via urothelial cell proliferation. Chen et al. proved that nicotine stimulates the cell proliferation of bladder cancer cells via the activation of Stat3 and ERK1/2 pathway (18). In addition, nicotine is closely related to tumor immunity and plays an important role in immunosuppression of both innate and adaptive immune systems (19). Tyagi et al. confirmed that nicotine-recruited N2 neutrophils induce MET (Mesenchymal-to-Epithelial Transition) conversion in breast cancer cells via the release of LCN2, thereby promoting cancer cell metastasis to the lungs (20). Growing evidence suggests that tobacco-smoking lung cancer patients exhibit elevated PD-L1 expression levels (21) and better responses to PD-1/PD-L1 inhibitors than nonsmokers (22). Moreover, nicotine mediates the up-regulation of PD-L1 expression and promotes the proliferation and migration of melanoma cell (23). Therefore, the immune status of tumor cell may be related with nicotine. In recent years, with the development of high-throughput sequencing technologies and the continuous expansion of databases, an increasing number of prognostic signatures have been constructed for predicting the tumor prognosis (24, 25). To investigate the influence of nicotine in bladder cancer, a robust NRS with four mRNAs was developed via machine learning algorithm to predict the prognosis and immunotherapy response of BLCA. The NRS demonstrated a reliable and accurate predictive capabilities for clinical outcomes and functioned as an independent prognostic factor in BLCA.

The signature contained four genes: AHNAK, MKRN1, SFXN4 and ZMYND8. Neuroblast differentiation-associated protein AHNAK is classified as a giant protein of 700 kDa and is localized in the plasma membrane, cytoplasm, and nucleus (26). Li et al. revealed that the increased expression of AHNAK contributes to the diagnosis of BLCA (16). It has been reported that AHNAK is significantly expressed in mast cells and is involved in TLR4-mediated mast cell activation signaling pathway (27). Studies have found that NAT10 promotes cisplatin resistance in bladder cancer by enhancing AHNAK-mediated DNA damage repair (28). Sideroflexin4 (SFXN4) belongs to the nuclear-encoded mitochondrial proteins family (29). Mutations in SFXN4 are associated with a mitochondrial disease, characterized by macrocytic anemia and a deficiency in complex I of the mitochondrial electron transport chain (30). Previous studies have demonstrated that SFXN4 may serve as a tumor promoter in ovarian cancer by the abnormal synthesis of Fe-S clusters and the active repair of DNA sequence (31). Zinc finger MYND-type containing 8 (ZMYND8), a chromatin reader protein recognizing histone H3 and H4, involves in the epigenetic regulation including modulation of chromatin integrity and DNA repair (32–35). Qiu et al. reported that ZMYND8 is regulated by E3 ubiquitin ligase FBXW7 and remarkably elevates the proliferation and invasiveness ability in BLCA cell (36). ZMYND8 is found to involve in macrophage-mediated inflammatory responses, and its knockout suppresses the expression of pro-inflammatory genes, such as Ccl2, Il1b, Il6, Cxcl10, and Nos2 (37). Wang et al. disclosed that ZMYND8 inhibits cytotoxic T cell-mediated anti-tumor immunity by suppressing the release of IFN-β from breast cancer cells, causing breast cancer cells to escape immune surveillance and promote tumorigenesis (38). Makorin ring finger protein 1 (MKRN1) is an E3 ubiquitin ligase involving the mediation of tumor progression and metabolic disorders by ubiquitinating substrates (39, 40). Due to the influence of environmental factor, expression level and target substrates, MKRN1 may have a tumor-promoter or suppressor function (41). Zhang et al. revealed that MKRN1 is overexpressed in colorectal cancer and promotes the TGF-β signaling activity via the ubiquitination and degradation of SNIP1 to induce EMT (Epithelial-to-Mesenchymal Transition) process of colorectal cancer cell (42). However, there is no research available on the biology role of MKRN1 in bladder cancer. Our study first reported that MKRN1 was a tumor-promoter factor in bladder cancer, and the knockdown of MKRN1 obviously suppressed the cell proliferation and migration in bladder cancer cell. Moreover, we observed that the bladder cancer cells with the treatment of nicotine exhibited enhanced proliferation and migration abilities. However, this effect was blocked down by loss of MKRN1, indicating that nicotine may promote the proliferation and migration of bladder cancer cells via MKRN1.

In our study, the NRS demonstrated good performance in predicting overall survival rate of bladder cancer patients. NRS functioned as an independent prognostic factor for OS. The results of ROC curve and C index chart showed that the AUC and C-index values of NRS were higher than tumor stage, suggesting the potential prognostic value of NRS. Stratified survival analysis indicated that NRS exhibited robust prognostic predictive capability across various clinical subgroups. Furthermore, our NRS suggested superior accuracy and effectiveness compared to published prognostic signatures for bladder cancer, as indicated by higher AUC and C-index values. This confirmed the robustness of our NRS using an integrated machine learning procedure.

Increasing evidences suggested that recent developments in immunotherapy have demonstrated increasing potential for the treatment of a variety of cancers, with particular promise observed in bladder cancer (43). In recent years, immune checkpoint inhibitors (ICIs), specifically anti-PD-1/PD-L1, have been applied for the treatment of metastatic urothelial carcinoma, and their role in bladder cancer is rapidly expanding. However, only a fraction of patients can benefit from immunotherapy due to low response rate and acquired resistance (44). Therefore, it is urgent to find biomarkers that predict response to immunotherapy. In this study, we assessed the performance of NRS from the perspective of prognosis and immunotherapy response in ICI-treated dataset. The BLCA patients with lower NRS scores had a better prognosis and higher response rate from immunotherapy, suggesting NRS may act as an indicator for predicting immunotherapy benefit. Previous studies have proven that immune checkpoint promotes tumor immune evasion by binding to the specific receptor on T cells, which inhibits T cell activation and proliferation, thereby reducing their anti-tumor activity (45). High expression of immune checkpoints may indicate that patients could respond to immunotherapy. In this study, we found that NRS scores were positivity associated with the expression levels of immune checkpoints. However, the survival rates and response rates to immunotherapy in the high-NRS group were not satisfactory. Numerous studies have demonstrated that the effectiveness of immunotherapy is not only affected by the level of immune checkpoint expression, but also hindered by presence of an immunosuppressive microenvironment. Therefore, we hypothesize that the unique TME in the high-NRS group may contribute to its poor response to immunotherapy.

Several studies indicated that TME is closely related to the initiation, progression, metastasis, and treatment response of bladder cancer (46, 47). TME is a complex entity comprising immune cells, stroma cells, blood vessels, extracellular matrix and cytokines (48). These cell components can collaborate in enabling tumor cells to evade immune surveillance and survive treatment (14). In the analysis of immune cell infiltration, CAF was predominantly abundant in high-NRS group. CAF is a key cell type within the TME and exhibits pro-tumor activities in various cancer types. It has been reported that inflammatory CAF is closely associated with poor prognosis in patients with bladder cancer (49). Recent studies have demonstrated that CAFs secrete a variety of cytokines and products that enhance the expression of immune checkpoint and diminish T cell activity, contributing to immune evasion and tumor progression (50). CAF-derived CXCL12 enhances the immune evasion of bladder cancer by inhibiting P62-mediated autophagic degradation of PD-L1, thereby promoting the growth of bladder cancer cells (51). Elevated levels of CAF may interfere with the functions of various immune cells, thereby reducing the effectiveness of immunotherapy (52). It has been verified that CAF can lead to reduced infiltration of CD8+ T cells within tumors and result in immune checkpoint blockade resistance (53). Therefore, the infiltration of CAF in the high-NRS group may account for the poor prognosis and failed immunotherapy in our study.

Functional enrichment analysis was performed to elucidate the potential mechanisms underlying the differences in prognosis, TME, immunotherapy response between high- and low-NRS groups in BLCA. Multiple signaling pathways were found to be associated with NRS, such as cytokine-cytokine receptor interactions, PI3k-Akt signaling pathway and IL-17 signaling pathway. Cytokine-cytokine receptor interactions has been reported to contribute to the progression of multiple tumors. Cytokines are pivotal in shaping the TME and have increasingly been utilized as monotherapy or in combination with other immunotherapy drugs for bladder cancer (54–56). Activation of PI3k-Akt signaling pathway is widely proven to be involved in the EMT process of bladder cancer (57). IL-17 is a cytokine known for its pro-inflammatory properties and antitumor immune responses. It has been highlighted in research for its substantial involvement in the initiation and progression of bladder cancer (58). Our analyses further demonstrate that NRS may mediate the progression of BLCA via these pathways.

There are some limitations in our study. The construction and validation of NRS are based on public database. Clinical samples and information are need to assess the effectiveness and feasibility of NRS. Moreover, the lack of *in vivo* experiments limits a more comprehensive exploration of the mechanism of MKRN1 in bladder cancer. Further research should focus on elucidating the role of MKRN1 in the progression of bladder cancer and its underlying molecular mechanisms.

## Conclusions

In conclusion, this study discloses a novel NRS via integrative machine learning analysis in BLCA. Our NRS provides valuable insights for predicting the prognosis and potential immunotherapy benefits in BLCA patients.

## Materials and methods

### Datasets sources

Bulk RNA-seq data of BLCA with survival time were acquired from TCGA and Gene Expression Omnibus (GEO) databases: TCGA-BLCA (n = 400) and GSE32894 (n = 308). TCGA-BLCA was randomly divided into TCGA-train and TCGA-test cohorts at a 1:1 ratio. TCGA-train cohort was used for the construction of signature. The validation cohorts for the analysis included TCGA-all, TCGA-test, and GSE32894. The IMvigor210 immunotherapy datasets (n = 298) was applied for the evaluation of immunotherapy benefits in signature. Single cell expression data of BLCA-GSE130001 was analyzed in online website TISCH. The selection of NRGs was collected from MSigDB (**Supplementary Table S1**).

### Identification of the nicotine metabolism clustering

Based on the expression of NRGs, consensus clustering was conducted to determine the optimal clusters via ConsensusClusterPlus package. The survival differences between clusters were evaluated via K-M survival curve. The CIBERSORT algorithm was employed to calculate the levels of immune cell infiltration in the identified clusters.

### Development and evaluation of a prognostic NRS

Differential expression analysis was performed among the clusters with the criterion of |log_2_ fold change (FC)| > 1 and P < 0.05. Then, intersected DEGs with remarkably different expression levels between BLCA and normal tissues were identified via limma package. Univariate Cox regression analysis was utilized to search for prognostic DEGs. To obtain an accurate and stable prognostic prediction signature, these prognostic DEGs were subjected to 100 integrative machine learning combination via 10 distinct machine learning algorithms (stepwise Cox, random survival forest [RSF], elastic network [Enet], supervised principal components [SuperPC], partial least squares regression for Cox [plsRcox], CoxBoost, survival support vector machine [survival-SVM], Lasso, Ridge and generalized boosted regression modeling [GBM]). The process of the integrative machine learning analysis was based on the R scripts (https://github.com/Zaoqu-Liu/IRLS) from previous studies. We constructed the NRS using following steps (1): Univariate Cox regression analysis was performed to search for prognostic genes in TCGA-train cohort. (2) Then, the prediction signature with 100 integrative machine learning combination was conducted in TCGA-train cohort. (3) All machine learning combination were detected in TCGA-test and GSE32894 cohorts. (4) C-index was calculated across all cohorts and the signature with the highest mean C-index was considered as the optimal NRS. Further detail about machine learning algorithms was shown in **Supplementary Methods**. The best cut-off value was calculated via the “surv_cutpoint” function within the survminer package to stratify BLCA patients into high- and low-NRS groups. K-M survival curve and ROC curve analyses were conducted to assess the predictive capability of NRS in predicting OS. Univariate and multivariate Cox analyses were employed to discover the important risk factor for the prognosis of BLCA. The predictive nomogram was constructed based on NRS and clinical characteristics using rms package.

### Immune infiltration analysis

To explore the correlation between NRS and TME, immune infiltration analysis was conducted via the XCELL, TIMER, QUANTISEQ, MCPCOUNTER, EPIC, CIBERSORT-ABS, and CIBERSORT algorithms. The ImmuneScore, StromalScore and ESTIMATE score for BLCA patients were computed via ESTIMATE package.

### Immunotherapy response analysis

To identify the correlation between NRS and immunotherapy response, the expression levels of immune checkpoints were estimated across different NRS groups. The predictive value of NRS in immunotherapy was further assessed in IMvigor210 cohort.

### Functional enrichment analysis

To elucidate the potential molecular mechanisms associated with NRS, functional enrichment analysis, including GO, KEGG and GSVA were performed between high- and low-NRS groups. GO and KEGG enrichment analyses were performed via clusterProfiler and org.Hs.eg.db packages. The GSVA values of KEGG pathway, calculated by GSVA package, were used to screen pathways highly correlated with NRS scores.

### Cell culture and transfection

Two human bladder cancer cell lines, 5637 and TCCSUP, were obtained from the American Type Culture Collection (ATCC). 5637 and TCCSUP cell lines were all maintained in RPMI-1640 medium containing 10% fetal bovine serum (FBS), 100 U/ml penicillin, and 100 μg/mL streptomycin in the cultivation environment of 5% CO^2^ and 37°C. MKRN1 inhibitors and its negative controls (NC) were produced from GenePharma company.

### CCK-8 assay

A CCK-8 kit was conducted for the measurement of proliferation abilities following the downregulation of MKRN1 in bladder cancer cells. In brief, the cells were inoculated into 96-well plates at a concentration of 3 × 10^3^ cells per well. At 0, 24, 48, 72 h after plating, 10 μL of CCK8 solution was added to each well for 3 h incubation in the dark. Finally, an enzyme labeling instrument was utilized to monitor the absorbance at a wavelength of 450 nm.

### Colony formation assay

Colony formation assay was executed to determine the cell colony formation rate. 5637 and TCCSUP cells were seeded into each well of a 6-well plate. Following a 14-day culture period, cell colonies were treated with 4% glutaraldehyde and subsequently stained using crystal violet.

### Cell migration assay

Transwell plates were used for the cell migration assay in accordance with the manufacturer’s protocol. Transfected cells were placed into Transwell filter membrane chambers with a serum-free medium. Meanwhile, 1 mL medium containing 20% FBS was supplemented into the lower chambers to serve as a chemoattractant. Following 36 h of incubation, the cells migrated to lower chambers were mixed with 4% paraformaldehyde for 20 min and were stained with crystal violet solution for 15 min.

### Statistical analysis

Statistical analyses and visualization were employed by R software and GraphPad Prism. Student’s t-test or one-way ANOVA was used to evaluate the differences between or among groups. Spearman’s test was employed to calculate the correlation coefficients. Data were presented as the mean ± standard deviation. P < 0.05 was deemed as statistically significant value.

## Data Availability

The raw data supporting the conclusions of this article will be made available by the authors, without undue reservation.
