# Supplementary material for: Identification and validation of the nicotine metabolism-related signature of bladder cancer by bioinformatics and machine learning
Source: Front Immunol. 2024 Dec 17;15:1465638. doi: 10.3389/fimmu.2024.1465638 (PMC11685211; doi:10.3389/fimmu.2024.1465638)
Supplement: Supplementary file 1 [file DataSheet1.zip › Original code.DOCX]

ibrary(limma)

setwd("G:")

rt=read.table("symbol.txt", header=T, sep="\t", check.names=F)

rt=as.matrix(rt)

rownames(rt)=rt[,1]

exp=rt[,2:ncol(rt)]

dimnames=list(rownames(exp), colnames(exp))

data=matrix(as.numeric(as.matrix(exp)), nrow=nrow(exp), dimnames=dimnames)

data=avereps(data)

data=data[rowMeans(data)>0,]

gene=read.table("gene.txt", header=F, sep="\t", check.names=F)

sameGene=intersect(as.vector(gene[,1]), rownames(data))

geneExp=data[sameGene,]

out=rbind(ID=colnames(geneExp),geneExp)

write.table(out,file="tcga.pyroptosisExp.txt",sep="\t",quote=F,col.names=F)

library(limma)

library(pheatmap)

expFile="tcga.pyroptosisExp.txt"

setwd("G:")

rt=read.table(expFile, header=T, sep="\t", check.names=F)

rt=as.matrix(rt)

rownames(rt)=rt[,1]

exp=rt[,2:ncol(rt)]

dimnames=list(rownames(exp), colnames(exp))

data=matrix(as.numeric(as.matrix(exp)), nrow=nrow(exp), dimnames=dimnames)

data=avereps(data)

group=sapply(strsplit(colnames(data),"\\-"), "[", 4)

group=sapply(strsplit(group,""), "[", 1)

group=gsub("2", "1", group)

conNum=length(group[group==1])

treatNum=length(group[group==0])

sampleType=c(rep(1,conNum), rep(2,treatNum))

sigVec=c()

outTab=data.frame()

for(i in rownames(data)){

if(sd(data[i,])<0.001){next}

wilcoxTest=wilcox.test(data[i,] ~ sampleType)

pvalue=wilcoxTest$p.value

if(pvalue<0.05){

Sig=ifelse(pvalue<0.001,"***",ifelse(pvalue<0.01,"**",ifelse(pvalue<0.05,"*","")))

sigVec=c(sigVec, paste0(i, Sig))

conGeneMeans=mean(data[i,1:conNum])

treatGeneMeans=mean(data[i,(conNum+1):ncol(data)])

logFC=log2(treatGeneMeans)-log2(conGeneMeans)

outTab=rbind(outTab,cbind(gene=i,conMean=conGeneMeans,treatMean=treatGeneMeans,logFC=logFC,pValue=pvalue))

}

}

write.table(outTab, file="diff.xls", sep="\t", row.names=F, quote=F)

write.table(outTab, file="diff.txt", sep="\t", row.names=F, quote=F)

exp=data[as.vector(outTab[,1]),]

expOut=rbind(ID=colnames(exp), exp)

write.table(expOut, file="diffGeneExp.txt", sep="\t", col.names=F, quote=F)

exp=log2(exp+0.1)

row.names(exp)=sigVec

Type=c(rep("Normal",conNum),rep("Tumor",treatNum))

names(Type)=colnames(data)

Type=as.data.frame(Type)

pdf(file="heatmap.pdf", width=9, height=8.5)

pheatmap(exp,

annotation=Type,

color = colorRampPalette(c(rep("blue",5), "white", rep("red",5)))(50),

cluster_cols =F,

cluster_rows =T,

scale="row",

show_colnames = F,

show_rownames = T,

fontsize = 8,

fontsize_row=8,

fontsize_col=8)

dev.off()

library(maftools)

setwd("G:")

geneRT=read.table("gene.txt", header=T, sep="\t", check.names=F, row.names=1)

gene=row.names(geneRT)

pdf(file="oncoplot.pdf", width=8, height=12)

maf=read.maf(maf="input.maf")

oncoplot(maf=maf, genes=gene, fontSize=0.5, draw_titv=T)

dev.off()

inputFile="cnvMatrix.txt"

setwd("G:")

rt=read.table(inputFile, header=T, sep="\t", check.names=F, row.names=1)

GAIN=rowSums(rt> 0)

LOSS=rowSums(rt< 0)

GAIN=GAIN/ncol(rt)*100

LOSS=LOSS/ncol(rt)*100

data=cbind(GAIN, LOSS)

data=data[order(data[,"GAIN"],decreasing = T),]

data.max = apply(data, 1, max)

pdf(file="CNVfreq.pdf", width=20, height=6)

cex=1.3

par(cex.lab=cex, cex.axis=cex, font.axis=2, las=1, xpd=T)

bar=barplot(data.max, col="grey80", border=NA,

xlab="", ylab="CNV.frequency(%)", space=1.5,

xaxt="n", ylim=c(0,1.2*max(data.max)))

points(bar,data[,"GAIN"], pch=20, col=2, cex=3)

points(bar,data[,"LOSS"], pch=20, col=3, cex=3)

legend("top", legend=c('GAIN','LOSS'), col=2:3, pch=20, bty="n", cex=2, ncol=2)

par(srt=45)

text(bar, par('usr')[3]-0.2, rownames(data), adj=1)

dev.off()

library("RCircos")

setwd("G:")

cytoBandIdeogram=read.table("refer.txt", header=T, sep="\t")

chr.exclude <- NULL

cyto.info <- cytoBandIdeogram

tracks.inside <- 5

tracks.outside <- 0

RCircos.Set.Core.Components(cyto.info, chr.exclude, tracks.inside, tracks.outside)

rcircos.params <- RCircos.Get.Plot.Parameters()

rcircos.params$text.size=1

rcircos.params$point.size=5

RCircos.Reset.Plot.Parameters(rcircos.params)

pdf(file="RCircos.pdf", width=8, height=8)

RCircos.Set.Plot.Area()

RCircos.Chromosome.Ideogram.Plot()

RCircos.Scatter.Data=read.table("Rcircos.scatter.txt", header=T, sep="\t", check.names=F)

data.col <- 4

track.num <- 1

side <- "in"

RCircos.Scatter.Plot(RCircos.Scatter.Data, data.col, track.num, side, by.fold=0.1)

RCircos.Gene.Label.Data=read.table("Rcircos.geneLabel.txt", header=T, sep="\t", check.names=F)

name.col <- 4

side <- "in"

track.num <- 2

RCircos.Gene.Connector.Plot(RCircos.Gene.Label.Data, track.num, side)

track.num <- 3

RCircos.Gene.Name.Plot(RCircos.Gene.Label.Data, name.col, track.num, side)

dev.off()

library(limma)

library(survival)

library(survminer)

expFile="prgGeneExp.txt"

cliFile="time.txt"

setwd("G:\\")

rt=read.table(expFile, header=T, sep="\t", check.names=F)

rt=as.matrix(rt)

rownames(rt)=rt[,1]

exp=rt[,2:ncol(rt)]

dimnames=list(rownames(exp), colnames(exp))

data=matrix(as.numeric(as.matrix(exp)), nrow=nrow(exp), dimnames=dimnames)

data=avereps(data)

data=data[rowMeans(data)>0,]

data=t(data)

rownames(data)=gsub("(.*?)\\_(.*?)", "\\2", rownames(data))

cli=read.table(cliFile, header=T, sep="\t", check.names=F, row.names=1)

cli$futime=cli$futime/365

sameSample=intersect(row.names(data), row.names(cli))

data=data[sameSample,]

cli=cli[sameSample,]

rt=cbind(cli, data)

outTab=data.frame()

km=c()

for(i in colnames(rt[,3:ncol(rt)])){

cox <- coxph(Surv(futime, fustat) ~ rt[,i], data = rt)

coxSummary = summary(cox)

coxP=coxSummary$coefficients[,"Pr(>|z|)"]

outTab=rbind(outTab,

cbind(id=i,

HR=coxSummary$conf.int[,"exp(coef)"],

HR.95L=coxSummary$conf.int[,"lower .95"],

HR.95H=coxSummary$conf.int[,"upper .95"],

pvalue=coxSummary$coefficients[,"Pr(>|z|)"])

)

data=rt[,c("futime", "fustat", i)]

colnames(data)=c("futime", "fustat", "gene")

res.cut=surv_cutpoint(data, time = "futime", event = "fustat", variables =c("gene"))

res.cat=surv_categorize(res.cut)

fit=survfit(Surv(futime, fustat) ~gene, data = res.cat)

#print(paste0(i, " ", res.cut$cutpoint[1]))

diff=survdiff(Surv(futime, fustat) ~gene,data =res.cat)

pValue=1-pchisq(diff$chisq, df=1)

km=c(km, pValue)

if(pValue<0.05){

if(pValue<0.001){

pValue="p<0.001"

}else{

pValue=paste0("p=",sprintf("%.03f",pValue))

}

surPlot=ggsurvplot(fit,

data=res.cat,

pval=pValue,

pval.size=6,

legend.title=i,

legend.labs=c("high","low"),

xlab="Time(years)",

ylab="Overall survival",

palette=c("red", "blue"),

break.time.by=1,

conf.int=F,

risk.table=F,

risk.table.title="",

risk.table.height=.25)

pdf(file=paste0("sur.", i, ".pdf"),onefile = FALSE,

width = 5,

height =4.5)

print(surPlot)

dev.off()

}

}

outTab=cbind(outTab, km)

write.table(outTab,file="uniCox.txt",sep="\t",row.names=F,quote=F)

library(igraph)

library(psych)

library(reshape2)

library(RColorBrewer)

GeneExpfile <- "prgGeneExp.txt"

Genefile <- "gene.txt"

Coxfile <- "uniCox.txt"

setwd("G:\\")

gene.group <- read.table(Genefile,header=T,sep="\t")

gene.exp <- read.table(GeneExpfile,header=T,sep="\t",row.names=1)

gene.cox <- read.table(Coxfile,header=T,sep="\t")

colnames(gene.group) <- c('id','group')

genelist <- intersect(gene.group$id, gene.cox$id)

genelist <- intersect(genelist, rownames(gene.exp))

gene.group <- gene.group[match(genelist,gene.group$id),]

gene.group <- gene.group[order(gene.group$group),]

gene.exp <- gene.exp[match(gene.group$id,rownames(gene.exp)),]

gene.cox <- gene.cox[match(gene.group$id,gene.cox$id),]

gene.cor <- corr.test(t(gene.exp))

gene.cor.cor <- gene.cor$r

gene.cor.pvalue <- gene.cor$p

gene.cor.cor[upper.tri(gene.cor.cor)] = NA

gene.cor.pvalue[upper.tri(gene.cor.pvalue)] = NA

gene.cor.cor.melt <- melt(gene.cor.cor) #gene1 \t gene2 \t cor

gene.cor.pvalue.melt <- melt(gene.cor.pvalue)

gene.melt <- data.frame(from = gene.cor.cor.melt$Var2,to=gene.cor.cor.melt$Var1,cor=gene.cor.cor.melt$value,pvalue=gene.cor.pvalue.melt$value)

gene.melt <- gene.melt[gene.melt$from!=gene.melt$to&!is.na(gene.melt$pvalue),,drop=F]

gene.edge <- gene.melt[gene.melt$pvalue<0.0001,,drop=F]

gene.edge$color <- ifelse(gene.edge$cor>0,'pink','#6495ED')

gene.edge$weight <- abs(gene.edge$cor)*6

gene.node <- gene.group

group.color <- colorRampPalette(brewer.pal(9, "Set1"))(length(unique(gene.node$group)))

gene.node$color <- group.color[as.numeric(as.factor(gene.node$group))]

gene.node$shape <- "circle"

gene.node$frame <- ifelse(gene.cox$HR>1,'purple',"green")

gene.node$pvalue <- gene.cox$pvalue

# pvalue size

pvalue.breaks <- c(0,0.0001,0.001,0.01,0.05,1)

pvalue.size <- c(16,14,12,10,8)

cutpvalue <- cut(gene.node$pvalue,breaks=pvalue.breaks)

gene.node$size <- pvalue.size[as.numeric(cutpvalue)]

nodefile <- "network.node.txt"

edgefile <- "network.edge.txt"

write.table(gene.node, nodefile, sep="\t", col.names=T, row.names=F, quote=F)

write.table(gene.edge, edgefile, sep="\t", col.names=T, row.names=F, quote=F)

node = read.table(nodefile, header=T, sep="\t", comment.char="")

edge = read.table(edgefile, header=T, sep="\t", comment.char="")

g = graph.data.frame(edge,directed = FALSE)

node = node[match(names(components(g)$membership),node$id),]

if(!is.na(match('color',colnames(node)))) V(g)$color = node$color

if(!is.na(match('size',colnames(node)))) V(g)$size = node$size

if(!is.na(match('shape',colnames(node)))) V(g)$shape = node$shape

if(!is.na(match('frame',colnames(node)))) V(g)$frame = node$frame

pdf(file="network.pdf", width=40, height=32)

par(mar=c(0,0,0,0))

layout(matrix(c(1,1,4,2,3,4),nc=2),height=c(4,4,2),width=c(8,3))

coord = layout_in_circle(g)

degree.x = acos(coord[,1])

degree.y = asin(coord[,2])

degree.alpha = c()

for(i in 1:length(degree.x)){

if(degree.y[i]<0) degree.alpha=c(degree.alpha,2*pi-degree.x[i]) else degree.alpha=c(degree.alpha,degree.x[i])

}

degree.cut.group = (0:8)/4*pi

degree.cut.group[1] = -0.0001

degree.cut = cut(degree.alpha,degree.cut.group)

degree.degree = c(-pi/4,-pi/4,-pi/2,-pi/2,pi/2,pi/2,pi/2,pi/4)

degree = degree.degree[as.numeric(degree.cut)]

values <- lapply(node$id,function(x)c(1,1))

V(g)$pie.color = lapply(1:nrow(node),function(x)c(node$color[x],node$frame[x]))

V(g)$frame = NA

plot(g,layout=layout_in_circle,vertex.shape="pie",vertex.pie=values,

vertex.label.cex=V(g)$lable.cex,edge.width = E(g)$weight,edge.arrow.size=0,

vertex.label.color=V(g)$color,vertex.frame.color=V(g)$frame,edge.color=E(g)$color,

vertex.label.cex=2,vertex.label.font=2,vertex.size=V(g)$size,edge.curved=0.4,

vertex.color=V(g)$color,vertex.label.dist=1,vertex.label.degree=degree)

# label.degree : zero means to the right; and pi means to the left; up is -pi/2 and down is pi/2; The default value is -pi/4

# label.dist If it is 0 then the label is centered on the vertex; If it is 1 then the label is displayed beside the vertex.

par(mar=c(0,0,0,0))

plot(1,type="n",xlab="",ylab="",axes=F)

groupinfo = unique(data.frame(group=node$group,color=node$color))

legend("left",legend=groupinfo$group,col=groupinfo$color,pch=16,bty="n",cex=3)

par(mar=c(0,0,0,0))

plot(1,type="n",xlab="",ylab="",axes=F)

legend("left",legend=c('Risk factors','Favorable factors'),col=c('purple','green'),pch=16,bty="n",cex=2.5)

par(mar=c(0,0,0,0))

plot(1,type="n",xlab="",axes=F,ylab="")

legend("top",legend=c('Postive correlation with P<0.0001','Negative correlation with P<0.0001'),lty=1,lwd=4,col=c('pink','#6495ED'),bty="n",cex=2.2)

legend('bottom',legend=c(0.0001,0.001,0.01,0.05,1),pch=16,pt.cex=c(1.6,1.4,1.2,1,0.8)*6,bty="n",ncol=5,cex=2.2,col="black",title="Cox test, pvalue")

dev.off()

library(limma)

library(survival)

library(ConsensusClusterPlus)

expFile="tcga.pyroptosisExp.txt"

cliFile="time.txt"

workDir="G:\\"

setwd(workDir)

data=read.table(expFile, header=T, sep="\t", check.names=F, row.names=1)

group=sapply(strsplit(colnames(data),"\\-"), "[", 4)

group=sapply(strsplit(group,""), "[", 1)

group=gsub("2", "1", group)

data=data[,group==0]

data=t(data)

rownames(data)=gsub("(.*?)\\-(.*?)\\-(.*?)\\-.*", "\\1\\-\\2\\-\\3", rownames(data))

data=avereps(data)

data=log2(data+1)

cli=read.table(cliFile,sep="\t",check.names=F,header=T,row.names=1)

sameSample=intersect(row.names(data),row.names(cli))

data=data[sameSample,]

cli=cli[sameSample,]

rt=cbind(cli,data)

sigGenes=c()

for(i in colnames(rt)[3:ncol(rt)]){

cox=coxph(Surv(futime, fustat) ~ rt[,i], data = rt)

coxSummary=summary(cox)

coxP=coxSummary$coefficients[,"Pr(>|z|)"]

if(coxP<0.05){ sigGenes=c(sigGenes,i) }

}

maxK=9

data=t(data[,sigGenes])

results=ConsensusClusterPlus(data,

maxK=maxK,

reps=50,

pItem=0.8,

pFeature=1,

title=workDir,

clusterAlg="pam",

distance="euclidean",

seed=123456,

plot="png")

clusterNum=2

Cluster=results[[clusterNum]][["consensusClass"]]

Cluster=as.data.frame(Cluster)

Cluster[,1]=paste0("C", Cluster[,1])

ClusterOut=rbind(ID=colnames(Cluster), Cluster)

write.table(ClusterOut, file="cluster.txt", sep="\t", quote=F, col.names=F)

library(survival)

library(survminer)

ClusterFile="cluster.txt"

cliFile="time.txt"

setwd("G:\\")

Cluster=read.table(ClusterFile, header=T, sep="\t", check.names=F, row.names=1)

cli=read.table(cliFile, header=T, sep="\t", check.names=F, row.names=1)

colnames(cli)=c("futime", "fustat")

cli$futime=cli$futime/365

sameSample=intersect(row.names(Cluster), row.names(cli))

rt=cbind(cli[sameSample,,drop=F], Cluster[sameSample,,drop=F])

length=length(levels(factor(rt$Cluster)))

diff=survdiff(Surv(futime, fustat) ~ Cluster, data = rt)

pValue=1-pchisq(diff$chisq, df=length-1)

if(pValue<0.001){

pValue="p<0.001"

}else{

pValue=paste0("p=",sprintf("%.03f",pValue))

}

fit <- survfit(Surv(futime, fustat) ~ Cluster, data = rt)

#print(surv_median(fit))

bioCol=c("#0066FF","#FF9900","#FF0000","#6E568C","#7CC767","#223D6C","#D20A13","#FFD121","#088247","#11AA4D")

bioCol=bioCol[1:length]

surPlot=ggsurvplot(fit,

data=rt,

conf.int=F,

pval=pValue,

pval.size=6,

legend.title="Cluster",

legend.labs=levels(factor(rt[,"Cluster"])),

legend = c(0.8, 0.8),

font.legend=10,

xlab="Time(years)",

break.time.by = 1,

palette = bioCol,

surv.median.line = "hv",

risk.table=T,

cumevents=F,

risk.table.height=.25)

pdf(file="survival.pdf",onefile = FALSE,width=7,height=5.5)

print(surPlot)

dev.off()

library(limma)

library(ggpubr)

cluFile="cluster.txt"

immFile="MCPcounter.result.txt"

setwd("G:\\")

immune=read.table(immFile, header=T, sep="\t", check.names=F, row.names=1)

immune=as.matrix(immune)

immune=t(immune)

cluster=read.table(cluFile, header=T, sep="\t", check.names=F, row.names=1)

sameSample=intersect(row.names(immune), row.names(cluster))

immune1=immune[sameSample,,drop=F]

cluster1=cluster[sameSample,,drop=F]

data=cbind(immune1, cluster1)

type=levels(factor(data[,"Cluster"]))

data$Cluster=factor(data$Cluster, levels=type)

comp=combn(type, 2)

my_comparisons=list()

for(i in 1:ncol(comp)){my_comparisons[[i]]<-comp[,i]}

bioCol=c("#0066FF","#FF0000","#FF9900","#6E568C","#7CC767","#223D6C","#D20A13","#FFD121","#088247","#11AA4D")

bioCol=bioCol[1:8]

for(i in colnames(data)[1:(ncol(data)-1)]){

data[,i][data[,i]>quantile(data[,i],0.99)]=quantile(data[,i],0.99)

violin=ggviolin(data, x="Cluster", y=i, fill = "Cluster",

xlab="", ylab=i,

legend.title="Cluster",

palette=bioCol,

pval=pValue,

pval.size=6,

add = "boxplot", add.params = list(fill="white"))+

stat_compare_means(comparisons = my_comparisons)

#stat_compare_means(comparisons = my_comparisons,symnum.args=list(cutpoints = c(0, 0.001, 0.01, 0.05, 1), symbols = c("***", "**", "*", "ns")),label = "p.signif")

pdf(file=paste0("violin.", i, ".pdf"), width=5, height=4.5)

print(violin)

dev.off()

}

library(limma)

library(pheatmap)

expFile="diffGeneExp.txt"

ClusterFile="cluster.txt"

cliFile="clinical.txt"

setwd("G:\\")

exp=read.table(expFile, header=T, sep="\t", check.names=F, row.names=1)

Cluster=read.table(ClusterFile, header=T, sep="\t", check.names=F, row.names=1)

cli=read.table(cliFile, header=T, sep="\t", check.names=F, row.names=1)

samSample=intersect(row.names(Cluster), row.names(cli))

cli=cli[samSample,,drop=F]

Cluster=Cluster[samSample,,drop=F]

Type=cbind(Cluster, cli)

Type=Type[order(Type$Cluster),,drop=F]

exp=exp[,row.names(Type)]

sigVec=c("Cluster")

for(clinical in colnames(Type[,2:ncol(Type)])){

data=Type[c("Cluster", clinical)]

colnames(data)=c("Cluster", "clinical")

data=data[(data[,"clinical"]!="unknow"),]

tableStat=table(data)

stat=chisq.test(tableStat)

pvalue=stat$p.value

Sig=ifelse(pvalue<0.001,"***",ifelse(pvalue<0.01,"**",ifelse(pvalue<0.05,"*","")))

sigVec=c(sigVec, paste0(clinical, Sig))

}

colnames(Type)=sigVec

colorList=list()

#Type=Type[apply(Type,1,function(x)any(is.na(match('unknow',x)))),,drop=F]

bioCol=c("#0066FF","#FF0000","#ed1299", "#0dbc21", "#246b93", "#cc8e12", "#d561dd",

"#6ad157", "#f7aa5d", "#9ed84e", "#39ba30", "#373bbf", "#a1ce4c", "#ef3bb6", "#d66551",

"#1a918f", "#ddd53e", "#ff66fc", "#2927c4", "#57e559" ,"#8e3af4" ,"#f9a270" ,"#22547f", "#db5e92",

"#4aef7b", "#e86502", "#99db27", "#e07233", "#8249aa","#cebb10", "#03827f", "#931635", "#ff523f",

"#edd05e", "#6f25e8", "#0dbc21", "#167275", "#280f7a", "#6373ed", "#5b910f" ,"#7b34c1" ,"#0cf29a" ,"#d80fc1",

"#dd27ce", "#07a301", "#391c82", "#2baeb5","#925bea", "#09f9f5", "#63ff4f")

j=0

for(cli in colnames(Type[,1:ncol(Type)])){

cliLength=length(levels(factor(Type[,cli])))

cliCol=bioCol[(j+1):(j+cliLength)]

j=j+cliLength

names(cliCol)=levels(factor(Type[,cli]))

if("unknow" %in% levels(factor(Type[,cli]))){

cliCol["unknow"]="grey75"}

colorList[[cli]]=cliCol

}

pdf("heatmap.pdf", width=8.5, height=6)

exp=log2(exp+0.01)

pheatmap(exp,

annotation=Type,

annotation_colors = colorList,

color = colorRampPalette(c(rep("blue",5), "white", rep("red",5)))(100),

cluster_cols =F,

cluster_rows =F,

scale="row",

show_colnames=F,

show_rownames=T,

fontsize=6,

fontsize_row=1,

fontsize_col=6)

dev.off()

library(limma)

library(sva)

tcgaExpFile="symbol.txt"

geoExpFile="geoMatrix.txt"

geneFile="diff.txt"

setwd("G:\\")

rt=read.table(tcgaExpFile, header=T, sep="\t", check.names=F)

rt=as.matrix(rt)

rownames(rt)=rt[,1]

exp=rt[,2:ncol(rt)]

dimnames=list(rownames(exp),colnames(exp))

tcga=matrix(as.numeric(as.matrix(exp)),nrow=nrow(exp),dimnames=dimnames)

tcga=avereps(tcga)

tcga=log2(tcga+1)

group=sapply(strsplit(colnames(tcga),"\\-"), "[", 4)

group=sapply(strsplit(group,""), "[", 1)

group=gsub("2", "1", group)

tcga=tcga[,group==0]

tcga=t(tcga)

rownames(tcga)=gsub("(.*?)\\-(.*?)\\-(.*?)\\-.*", "\\1\\-\\2\\-\\3", rownames(tcga))

tcga=t(avereps(tcga))

rt=read.table(geoExpFile, header=T, sep="\t", check.names=F)

rt=as.matrix(rt)

rownames(rt)=rt[,1]

exp=rt[,2:ncol(rt)]

dimnames=list(rownames(exp),colnames(exp))

geo=matrix(as.numeric(as.matrix(exp)),nrow=nrow(exp),dimnames=dimnames)

geo=avereps(geo)

qx=as.numeric(quantile(geo, c(0, 0.25, 0.5, 0.75, 0.99, 1.0), na.rm=T))

LogC=( (qx[5]>100) || ( (qx[6]-qx[1])>50 && qx[2]>0) )

if(LogC){

geo[geo<0]=0

geo=log2(geo+1)}

geo=normalizeBetweenArrays(geo)

sameGene=intersect(row.names(tcga),row.names(geo))

tcgaOut=tcga[sameGene,]

geoOut=geo[sameGene,]

all=cbind(tcgaOut,geoOut)

batchType=c(rep(1,ncol(tcgaOut)),rep(2,ncol(geoOut)))

outTab=ComBat(all, batchType, par.prior=TRUE)

tcgaOut=outTab[,colnames(tcgaOut)]

tcgaOut[tcgaOut<0]=0

geoOut=outTab[,colnames(geoOut)]

geoOut[geoOut<0]=0

tcgaTab=rbind(ID=colnames(tcgaOut), tcgaOut)

write.table(tcgaTab, file="TCGA.normalize.txt", sep="\t", quote=F, col.names=F)

geoTab=rbind(ID=colnames(geoOut), geoOut)

write.table(geoTab,file="GEO.normalize.txt",sep="\t",quote=F,col.names=F)

gene=read.table(geneFile, header=T, sep="\t", check.names=F)

sameGene=intersect(as.vector(gene[,1]), rownames(tcgaOut))

tcgaShareExp=tcgaOut[sameGene,]

geoShareExp=geoOut[sameGene,]

tcgaShareExp=rbind(ID=colnames(tcgaShareExp),tcgaShareExp)

write.table(tcgaShareExp,file="TCGA.share.txt",sep="\t",quote=F,col.names=F)

geoShareExp=rbind(ID=colnames(geoShareExp),geoShareExp)

write.table(geoShareExp,file="GEO.share.txt",sep="\t",quote=F,col.names=F)

library(limma)

expFile="tcga.share.txt"

cliFile="time.txt"

setwd("G:\\")

rt=read.table(expFile, header=T, sep="\t", check.names=F)

rt=as.matrix(rt)

rownames(rt)=rt[,1]

exp=rt[,2:ncol(rt)]

dimnames=list(rownames(exp),colnames(exp))

data=matrix(as.numeric(as.matrix(exp)),nrow=nrow(exp),dimnames=dimnames)

data=avereps(data)

data=data[rowMeans(data)>0,]

data=t(data)

cli=read.table(cliFile,sep="\t",check.names=F,header=T,row.names=1)

sameSample=intersect(row.names(data),row.names(cli))

data=data[sameSample,]

cli=cli[sameSample,]

out=cbind(cli,data)

out=cbind(id=row.names(out),out)

write.table(out,file="tcga.expTime.txt",sep="\t",row.names=F,quote=F)

library(survival)

coxPfilter=0.05

inputFile="tcga.expTime.txt"

setwd("")

rt=read.table(inputFile, header=T, sep="\t", check.names=F, row.names=1)

rt$futime=rt$futime/365

outTab=data.frame()

sigGenes=c("futime","fustat")

for(i in colnames(rt[,3:ncol(rt)])){

cox <- coxph(Surv(futime, fustat) ~ rt[,i], data = rt)

coxSummary = summary(cox)

coxP=coxSummary$coefficients[,"Pr(>|z|)"]

if(coxP<coxPfilter){

sigGenes=c(sigGenes,i)

outTab=rbind(outTab,

cbind(id=i,

HR=coxSummary$conf.int[,"exp(coef)"],

HR.95L=coxSummary$conf.int[,"lower .95"],

HR.95H=coxSummary$conf.int[,"upper .95"],

pvalue=coxSummary$coefficients[,"Pr(>|z|)"])

)

}

}

write.table(outTab,file="tcga.uniCox.txt",sep="\t",row.names=F,quote=F)

uniSigExp=rt[,sigGenes]

uniSigExp=cbind(id=row.names(uniSigExp),uniSigExp)

write.table(uniSigExp,file="tcga.uniSigExp.txt",sep="\t",row.names=F,quote=F)

bioForest=function(coxFile=null,forestFile=null,forestCol=null){

rt <- read.table(coxFile,header=T,sep="\t",row.names=1,check.names=F)

gene <- rownames(rt)

hr <- sprintf("%.3f",rt$"HR")

hrLow <- sprintf("%.3f",rt$"HR.95L")

hrHigh <- sprintf("%.3f",rt$"HR.95H")

Hazard.ratio <- paste0(hr,"(",hrLow,"-",hrHigh,")")

pVal <- ifelse(rt$pvalue<0.001, "<0.001", sprintf("%.3f", rt$pvalue))

height=nrow(rt)/12.5+5

pdf(file=forestFile, width = 7,height = height)

n <- nrow(rt)

nRow <- n+1

ylim <- c(1,nRow)

layout(matrix(c(1,2),nc=2),width=c(3,2.5))

xlim = c(0,3)

par(mar=c(4,2.5,2,1))

plot(1,xlim=xlim,ylim=ylim,type="n",axes=F,xlab="",ylab="")

text.cex=0.8

text(0,n:1,gene,adj=0,cex=text.cex)

text(1.5-0.5*0.2,n:1,pVal,adj=1,cex=text.cex);text(1.5-0.5*0.2,n+1,'pvalue',cex=text.cex,font=2,adj=1)

text(3,n:1,Hazard.ratio,adj=1,cex=text.cex);text(3,n+1,'Hazard ratio',cex=text.cex,font=2,adj=1,)

par(mar=c(4,1,2,1),mgp=c(2,0.5,0))

xlim = c(0,max(as.numeric(hrLow),as.numeric(hrHigh)))

plot(1,xlim=xlim,ylim=ylim,type="n",axes=F,ylab="",xaxs="i",xlab="Hazard ratio")

arrows(as.numeric(hrLow),n:1,as.numeric(hrHigh),n:1,angle=90,code=3,length=0.05,col="darkblue",lwd=2.5)

abline(v=1,col="black",lty=2,lwd=2)

boxcolor = ifelse(as.numeric(hr) > 1, forestCol[1], forestCol[2])

points(as.numeric(hr), n:1, pch = 15, col = boxcolor, cex=1.6)

axis(1)

dev.off()

}

bioForest(coxFile="tcga.uniCox.txt",forestFile="forest.pdf",forestCol=c("red","green"))

library("glmnet")

library("survival")

coxSigFile="tcga.uniSigExp.txt"

geoFile="geo.expTime.txt"

setwd("G:\\")

rt=read.table(coxSigFile, header=T, sep="\t", check.names=F, row.names=1)

geo=read.table(geoFile, header=T, sep="\t", check.names=F, row.names=1)

sameGene=intersect(colnames(rt)[3:ncol(rt)], colnames(geo)[3:ncol(geo)])

rt=rt[,c("futime","fustat",sameGene)]

rt$futime[rt$futime<=0]=0.003

x=as.matrix(rt[,c(3:ncol(rt))])

y=data.matrix(Surv(rt$futime, rt$fustat))

fit=glmnet(x, y, family="cox", maxit=2000)

pdf("lasso.lambda.pdf")

plot(fit, xvar = "lambda", label = TRUE)

dev.off()

cvfit=cv.glmnet(x, y, family="cox", maxit=1000)

pdf("lasso.cvfit.pdf")

plot(cvfit)

abline(v=log(c(cvfit$lambda.min,cvfit$lambda.1se)),lty="dashed")

dev.off()

coef=coef(fit, s=cvfit$lambda.min)

index=which(coef != 0)

actCoef=coef[index]

lassoGene=row.names(coef)[index]

geneCoef=cbind(Gene=lassoGene, Coef=actCoef)

write.table(geneCoef, file="lasso.geneCoef.txt", sep="\t", quote=F, row.names=F)

trainFinalGeneExp=rt[,lassoGene]

myFun=function(x){crossprod(as.numeric(x),actCoef)}

trainScore=apply(trainFinalGeneExp,1,myFun)

outCol=c("futime","fustat",lassoGene)

risk=as.vector(ifelse(trainScore>median(trainScore),"high","low"))

outTab=cbind(rt[,outCol],riskScore=as.vector(trainScore),risk)

write.table(cbind(id=rownames(outTab),outTab),file="trainRisk.txt",sep="\t",quote=F,row.names=F)

rt=read.table(geoFile, header=T, sep="\t", check.names=F, row.names=1)

rt$futime=rt$futime/365

testFinalGeneExp=rt[,lassoGene]

testScore=apply(testFinalGeneExp,1,myFun)

outCol=c("futime","fustat",lassoGene)

risk=as.vector(ifelse(testScore>median(trainScore),"high","low"))

outTab=cbind(rt[,outCol],riskScore=as.vector(testScore),risk)

write.table(cbind(id=rownames(outTab),outTab),file="testRisk.txt",sep="\t",quote=F,row.names=F)

library(survival)

library(survminer)

library(timeROC)

setwd("G:\\")

bioROC=function(inputFile=null, rocFile=null){

rt=read.table(inputFile, header=T, sep="\t", check.names=F)

ROC_rt=timeROC(T=rt$futime, delta=rt$fustat,

marker=rt$riskScore, cause=1,

weighting='aalen',

times=c(1,3,5), ROC=TRUE)

pdf(file=rocFile,width=5,height=5)

plot(ROC_rt,time=1,col='green',title=FALSE,lwd=2)

plot(ROC_rt,time=3,col='blue',add=TRUE,title=FALSE,lwd=2)

plot(ROC_rt,time=5,col='red',add=TRUE,title=FALSE,lwd=2)

legend('bottomright',

c(paste0('AUC at 1 years: ',sprintf("%.03f",ROC_rt$AUC[1])),

paste0('AUC at 3 years: ',sprintf("%.03f",ROC_rt$AUC[2])),

paste0('AUC at 5 years: ',sprintf("%.03f",ROC_rt$AUC[3]))),

col=c("green","blue","red"),lwd=2,bty = 'n')

dev.off()

}

bioROC(inputFile="trainRisk.txt", rocFile="train.ROC.pdf")

bioROC(inputFile="testRisk.txt", rocFile="test.ROC.pdf")

library(pheatmap)

setwd("G:\\")

bioRiskPlot=function(inputFile=null, riskScoreFile=null, survStatFile=null){

rt=read.table(inputFile, header=T, sep="\t", check.names=F, row.names=1)

rt$riskScore[rt$riskScore>quantile(rt$riskScore,0.99)]=quantile(rt$riskScore,0.99)

rt$risk=factor(rt$risk, levels=c("low", "high"))

rt=rt[order(rt$riskScore),]

riskClass=rt[,"risk"]

lowLength=length(riskClass[riskClass=="low"])

highLength=length(riskClass[riskClass=="high"])

lowMax=max(rt$riskScore[riskClass=="low"])

line=rt[,"riskScore"]

pdf(file=riskScoreFile, width=6, height=5)

plot(line, type="p", pch=20,

xlab="Patients (increasing risk socre)", ylab="Risk score",

col=c(rep("blue",lowLength),rep("red",highLength)) )

abline(h=lowMax,v=lowLength,lty=2)

legend("topleft", c("High risk", "Low Risk"),bty="n",pch=19,col=c("red","blue"),cex=1.2)

dev.off()

color=as.vector(rt$fustat)

color[color==1]="red"

color[color==0]="blue"

pdf(file=survStatFile, width=6, height=5)

plot(rt$futime, pch=19,

xlab="Patients (increasing risk socre)", ylab="Survival time (years)",

col=color)

legend("topleft", c("Dead", "Alive"),bty="n",pch=19,col=c("red","blue"),cex=1.2)

abline(v=lowLength,lty=2)

dev.off()

}

bioRiskPlot(inputFile="trainRisk.txt",

riskScoreFile="train.riskScore.pdf",

survStatFile="train.survStat.pdf")

bioRiskPlot(inputFile="testRisk.txt",

riskScoreFile="test.riskScore.pdf",

survStatFile="test.survStat.pdf")

library(Rtsne)

library(ggplot2)

setwd("G:\\")

bioPCA=function(inputFile=null, pcaFile=null, tsneFile=null){

rt=read.table(inputFile, header=T, sep="\t", check.names=F, row.names=1)

data=rt[c(3:(ncol(rt)-2))]

risk=rt[,"risk"]

data.pca=prcomp(data, scale. = TRUE)

pcaPredict=predict(data.pca)

PCA = data.frame(PC1 = pcaPredict[,1], PC2 = pcaPredict[,2],risk=risk)

pdf(file=pcaFile, height=4.5, width=5.5)

p=ggplot(data = PCA, aes(PC1, PC2)) + geom_point(aes(color = risk)) +

scale_colour_manual(name="Risk", values =c("red", "blue"))+

theme_bw()+

theme(plot.margin=unit(rep(1.5,4),'lines'))+

theme(panel.grid.major = element_blank(), panel.grid.minor = element_blank())

print(p)

dev.off()

tsneOut=Rtsne(data, dims=2, perplexity=10, verbose=F, max_iter=500,check_duplicates=F)

tsne=data.frame(tSNE1 = tsneOut$Y[,1], tSNE2 = tsneOut$Y[,2],risk=risk)

pdf(file=tsneFile, height=4.5, width=5.5)

p=ggplot(data = tsne, aes(tSNE1, tSNE2)) + geom_point(aes(color = risk)) +

scale_colour_manual(name="Risk", values =c("red", "blue"))+

theme_bw()+

theme(plot.margin=unit(rep(1.5,4),'lines'))+

theme(panel.grid.major = element_blank(), panel.grid.minor = element_blank())

print(p)

dev.off()

}

bioPCA(inputFile="trainRisk.txt", pcaFile="train.PCA.pdf", tsneFile="train.t-SNE.pdf")

bioPCA(inputFile="testRisk.txt", pcaFile="test.PCA.pdf", tsneFile="test.t-SNE.pdf")

library(survival)

setwd("G:\\")

bioForest=function(coxFile=null, forestFile=null, forestCol=null){

rt <- read.table(coxFile,header=T,sep="\t",row.names=1,check.names=F)

gene <- rownames(rt)

hr <- sprintf("%.3f",rt$"HR")

hrLow <- sprintf("%.3f",rt$"HR.95L")

hrLow[hrLow<0.001]=0.001

hrHigh <- sprintf("%.3f",rt$"HR.95H")

Hazard.ratio <- paste0(hr,"(",hrLow,"-",hrHigh,")")

pVal <- ifelse(rt$pvalue<0.001, "<0.001", sprintf("%.3f", rt$pvalue))

pdf(file=forestFile, width=6.5, height=4.8)

n <- nrow(rt)

nRow <- n+1

ylim <- c(1,nRow)

layout(matrix(c(1,2),nc=2),width=c(3,2.5))

xlim = c(0,3)

par(mar=c(4,2.5,2,1))

plot(1,xlim=xlim,ylim=ylim,type="n",axes=F,xlab="",ylab="")

text.cex=0.8

text(0,n:1,gene,adj=0,cex=text.cex)

text(1.5-0.5*0.2,n:1,pVal,adj=1,cex=text.cex);text(1.5-0.5*0.2,n+1,'pvalue',cex=text.cex,adj=1)

text(3,n:1,Hazard.ratio,adj=1,cex=text.cex);text(3,n+1,'Hazard ratio',cex=text.cex,adj=1,)

par(mar=c(4,1,2,1),mgp=c(2,0.5,0))

LOGindex=2

hrLow = log(as.numeric(hrLow),LOGindex)

hrHigh = log(as.numeric(hrHigh),LOGindex)

hr = log(as.numeric(hr),LOGindex)

xlim = c(floor(min(hrLow,hrHigh)),ceiling(max(hrLow,hrHigh)))

plot(1,xlim=xlim,ylim=ylim,type="n",axes=F,ylab="",xaxs="i",xlab="Hazard ratio")

arrows(as.numeric(hrLow),n:1,as.numeric(hrHigh),n:1,angle=90,code=3,length=0.05,col="darkblue",lwd=2.5)

abline(v=log(1,LOGindex),col="black",lty=2,lwd=2)

boxcolor = ifelse(as.numeric(hr) > log(1,LOGindex), forestCol, forestCol)

points(as.numeric(hr), n:1, pch = 15, col = boxcolor, cex=2)

a1 = axis(1,labels=F,tick=F)

axis(1,a1,LOGindex^a1)

dev.off()

}

indep=function(riskFile=null,cliFile=null,uniOutFile=null,multiOutFile=null,uniForest=null,multiForest=null){

risk=read.table(riskFile, header=T, sep="\t", check.names=F, row.names=1) #¶ÁÈ¡·çÏÕÎÄ¼þ

cli=read.table(cliFile, header=T, sep="\t", check.names=F, row.names=1) #¶ÁÈ¡ÁÙ´²ÎÄ¼þ

sameSample=intersect(row.names(cli),row.names(risk))

risk=risk[sameSample,]

cli=cli[sameSample,]

rt=cbind(futime=risk[,1], fustat=risk[,2], cli, riskScore=risk[,(ncol(risk)-1)])

uniTab=data.frame()

for(i in colnames(rt[,3:ncol(rt)])){

cox <- coxph(Surv(futime, fustat) ~ rt[,i], data = rt)

coxSummary = summary(cox)

uniTab=rbind(uniTab,

cbind(id=i,

HR=coxSummary$conf.int[,"exp(coef)"],

HR.95L=coxSummary$conf.int[,"lower .95"],

HR.95H=coxSummary$conf.int[,"upper .95"],

pvalue=coxSummary$coefficients[,"Pr(>|z|)"])

)

}

write.table(uniTab,file=uniOutFile,sep="\t",row.names=F,quote=F)

bioForest(coxFile=uniOutFile, forestFile=uniForest, forestCol="green")

uniTab=uniTab[as.numeric(uniTab[,"pvalue"])<0.05,]

rt1=rt[,c("futime", "fustat", as.vector(uniTab[,"id"]))]

multiCox=coxph(Surv(futime, fustat) ~ ., data = rt1)

multiCoxSum=summary(multiCox)

multiTab=data.frame()

multiTab=cbind(

HR=multiCoxSum$conf.int[,"exp(coef)"],

HR.95L=multiCoxSum$conf.int[,"lower .95"],

HR.95H=multiCoxSum$conf.int[,"upper .95"],

pvalue=multiCoxSum$coefficients[,"Pr(>|z|)"])

multiTab=cbind(id=row.names(multiTab),multiTab)

write.table(multiTab,file=multiOutFile,sep="\t",row.names=F,quote=F)

bioForest(coxFile=multiOutFile, forestFile=multiForest, forestCol="red")

}

indep(riskFile="trainRisk.txt",

cliFile="clinical.txt",

uniOutFile="uniCox.txt",

multiOutFile="multiCox.txt",

uniForest="uniForest.pdf",

multiForest="multiForest.pdf")

library(survival)

library(regplot)

library(rms)

riskFile="risk.TCGAall.txt"

cliFile="clinical.txt"

setwd("G:\\")

risk=read.table(riskFile, header=T, sep="\t", check.names=F, row.names=1)

cli=read.table(cliFile, header=T, sep="\t", check.names=F, row.names=1)

cli=cli[apply(cli,1,function(x)any(is.na(match('unknow',x)))),,drop=F]

cli$Age=as.numeric(cli$Age)

samSample=intersect(row.names(risk), row.names(cli))

risk1=risk[samSample,,drop=F]

cli=cli[samSample,,drop=F]

rt=cbind(risk1[,c("futime", "fustat", "Risk")], cli)

res.cox=coxph(Surv(futime, fustat) ~ . , data = rt)

nom1=regplot(res.cox,

plots = c("density", "boxes"),

clickable=F,

title="",

points=TRUE,

droplines=TRUE,

observation=rt[1,],

rank="sd",

failtime = c(1,3,5),

prfail = F)

nomoRisk=predict(res.cox, data=rt, type="risk")

rt=cbind(risk1, Nomogram=nomoRisk)

outTab=rbind(ID=colnames(rt), rt)

write.table(outTab, file="nomoRisk.txt", sep="\t", col.names=F, quote=F)

pdf(file="calibration.pdf", width=5, height=5)

f <- cph(Surv(futime, fustat) ~ Nomogram, x=T, y=T, surv=T, data=rt, time.inc=1)

cal <- calibrate(f, cmethod="KM", method="boot", u=1, m=(nrow(rt)/3), B=1000)

plot(cal, xlim=c(0,1), ylim=c(0,1),

xlab="Nomogram-predicted OS (%)", ylab="Observed OS (%)", lwd=1.5, col="green", sub=F)

f <- cph(Surv(futime, fustat) ~ Nomogram, x=T, y=T, surv=T, data=rt, time.inc=3)

cal <- calibrate(f, cmethod="KM", method="boot", u=3, m=(nrow(rt)/3), B=1000)

plot(cal, xlim=c(0,1), ylim=c(0,1), xlab="", ylab="", lwd=1.5, col="blue", sub=F, add=T)

f <- cph(Surv(futime, fustat) ~ Nomogram, x=T, y=T, surv=T, data=rt, time.inc=5)

cal <- calibrate(f, cmethod="KM", method="boot", u=5, m=(nrow(rt)/3), B=1000)

plot(cal, xlim=c(0,1), ylim=c(0,1), xlab="", ylab="", lwd=1.5, col="red", sub=F, add=T)

legend('bottomright', c('1-year', '3-year', '5-year'),

col=c("green","blue","red"), lwd=1.5, bty = 'n')

dev.off()

library(survival)

library(survminer)

library(timeROC)

library(ggDCA)

predictTime=1

riskFile="nomoRisk.txt"

cliFile="clinical.txt"

setwd("")

risk=read.table(riskFile, header=T, sep="\t", check.names=F, row.names=1)

cli=read.table(cliFile, header=T, sep="\t", check.names=F, row.names=1)

samSample=intersect(row.names(risk), row.names(cli))

risk1=risk[samSample,,drop=F]

cli1=cli[samSample,,drop=F]

data=cbind(risk1, cli1)

rt=cbind(risk1[,c("futime","fustat","Risk","Nomogram")], cli1)

rt[,"Age"]=ifelse(rt[,"Age"]>65, 1, 0)

rt[,"Nomogram"]=ifelse(rt[,"Nomogram"]>median(rt[,"Nomogram"]), 1, 0)

Nomogram<-coxph(Surv(futime,fustat)~Nomogram,rt)

Risk<-coxph(Surv(futime,fustat)~Risk,rt)

Age<-coxph(Surv(futime,fustat)~Age,rt)

Gender<-coxph(Surv(futime,fustat)~Gender,rt)

Grade<-coxph(Surv(futime,fustat)~Grade,rt)

Stage<-coxph(Surv(futime,fustat)~Stage,rt)

pdf(file="DCA.pdf", width=6.5, height=5.2)

d_train=dca(Nomogram,Risk,Age,Gender,Grade,Stage, times=predictTime)

ggplot(d_train, linetype=1)

dev.off()

rt=cbind(risk1[,c("futime","fustat","riskScore","Nomogram")], cli1)

aucText=c()

bioCol=rainbow(ncol(rt)-1, s=0.9, v=0.9)

pdf(file="cliROC.pdf", width=6, height=6)

i=3

ROC_rt=timeROC(T=risk$futime,

delta=risk$fustat,

marker=risk$riskScore, cause=1,

weighting='aalen',

times=c(predictTime),ROC=TRUE)

plot(ROC_rt, time=predictTime, col=bioCol[i-2], title=FALSE, lwd=2)

aucText=c(paste0("Risk", ", AUC=", sprintf("%.3f",ROC_rt$AUC[2])))

abline(0,1)

for(i in 4:ncol(rt)){

ROC_rt=timeROC(T=rt$futime,

delta=rt$fustat,

marker=rt[,i], cause=1,

weighting='aalen',

times=c(predictTime),ROC=TRUE)

plot(ROC_rt, time=predictTime, col=bioCol[i-2], title=FALSE, lwd=2, add=TRUE)

aucText=c(aucText, paste0(colnames(rt)[i],", AUC=",sprintf("%.3f",ROC_rt$AUC[2])))

}

legend("bottomright", aucText,lwd=2,bty="n",col=bioCol[1:(ncol(rt)-1)])

dev.off()

library(limma)

library(pheatmap)

riskFile="trainRisk.txt"

cliFile="clinical.txt"

setwd("G:\\")

cli=read.table(cliFile, header=T, sep="\t", check.names=F, row.names=1)

risk=read.table(riskFile, header=T, sep="\t", check.names=F, row.names=1)

risk$risk=factor(risk$risk, levels=c("low", "high"))

samSample=intersect(row.names(risk), row.names(cli))

cli=cli[samSample,,drop=F]

risk=risk[samSample,,drop=F]

data=cbind(risk, cli)

data=data[order(data$riskScore),,drop=F]

Type=data[,(ncol(risk):ncol(data))]

exp=data[,(3:(ncol(risk)-2))]

sigVec=c("risk")

for(clinical in colnames(Type[,2:ncol(Type)])){

data=Type[c("risk", clinical)]

colnames(data)=c("risk", "clinical")

data=data[(data[,"clinical"]!="unknow"),]

tableStat=table(data)

stat=chisq.test(tableStat)

pvalue=stat$p.value

Sig=ifelse(pvalue<0.001,"***",ifelse(pvalue<0.01,"**",ifelse(pvalue<0.05,"*","")))

sigVec=c(sigVec, paste0(clinical, Sig))

}

colnames(Type)=sigVec

colorList=list()

#Type=Type[apply(Type,1,function(x)any(is.na(match('unknow',x)))),,drop=F]

bioCol=c("#0066FF","#FF0000","#ed1299", "#0dbc21", "#246b93", "#cc8e12", "#d561dd",

"#6ad157", "#f7aa5d", "#9ed84e", "#39ba30", "#373bbf", "#a1ce4c", "#ef3bb6", "#d66551",

"#1a918f", "#ddd53e", "#ff66fc", "#2927c4", "#57e559" ,"#8e3af4" ,"#f9a270" ,"#22547f", "#db5e92",

"#4aef7b", "#e86502", "#99db27", "#e07233", "#8249aa","#cebb10", "#03827f", "#931635", "#ff523f",

"#edd05e", "#6f25e8", "#0dbc21", "#167275", "#280f7a", "#6373ed", "#5b910f" ,"#7b34c1" ,"#0cf29a" ,"#d80fc1",

"#dd27ce", "#07a301", "#391c82", "#2baeb5","#925bea", "#09f9f5", "#63ff4f")

j=0

for(cli in colnames(Type[,1:ncol(Type)])){

cliLength=length(levels(factor(Type[,cli])))

cliCol=bioCol[(j+1):(j+cliLength)]

j=j+cliLength

names(cliCol)=levels(factor(Type[,cli]))

if("unknow" %in% levels(factor(Type[,cli]))){

cliCol["unknow"]="grey75"}

colorList[[cli]]=cliCol

}

pdf("heatmap.pdf", width=9, height=6)

pheatmap(t(exp),

annotation=Type,

annotation_colors = colorList,

color = colorRampPalette(c(rep("blue",3), "white", rep("red",3)))(100),

cluster_cols =F,

cluster_rows =F,

scale="row",

show_colnames=F,

show_rownames=T,

fontsize=6,

fontsize_row=7,

fontsize_col=6)

dev.off()

library("clusterProfiler")

library("org.Hs.eg.db")

library("enrichplot")

library("ggplot2")

pvalueFilter=0.05

qvalueFilter=0.05

setwd("G:\\")

rt=read.table("id.txt",sep="\t",header=T,check.names=F)

rt=rt[is.na(rt[,"entrezID"])==F,]

gene=rt$entrezID

geneFC=2^rt$logFC

names(geneFC)=gene

colorSel="qvalue"

if(qvalueFilter>0.05){

colorSel="pvalue"

}

kk=enrichGO(gene = gene,OrgDb = org.Hs.eg.db, pvalueCutoff =1, qvalueCutoff = 1, ont="all", readable =T)

GO=as.data.frame(kk)

GO=GO[(GO$pvalue<pvalueFilter & GO$qvalue<qvalueFilter),]

write.table(GO,file="GO.txt",sep="\t",quote=F,row.names = F)

showNum=10

if(nrow(GO)<30){

showNum=nrow(GO)

}

pdf(file="barplot.pdf",width = 9,height = 11)

bar=barplot(kk, drop = TRUE, showCategory =showNum,split="ONTOLOGY",color = colorSel) + facet_grid(ONTOLOGY~., scale='free')

print(bar)

dev.off()

pdf(file="bubble.pdf",width = 9,height = 11)

bub=dotplot(kk,showCategory = showNum, orderBy = "GeneRatio",split="ONTOLOGY", color = colorSel) + facet_grid(ONTOLOGY~., scale='free')

print(bub)

dev.off()

pdf(file="circos.pdf",width = 9,height = 7)

cnet=cnetplot(kk, foldChange=geneFC, showCategory = 5, circular = TRUE, colorEdge = TRUE)

print(cnet)

dev.off()

library(limma)

library(org.Hs.eg.db)

library(clusterProfiler)

library(enrichplot)

expFile="symbol.txt"

riskFile="risk.TCGAall.txt"

gmtFile="c2.cp.kegg.v7.4.symbols.gmt"

setwd("G:\\")

rt=read.table(expFile, header=T, sep="\t", check.names=F)

rt=as.matrix(rt)

rownames(rt)=rt[,1]

exp=rt[,2:ncol(rt)]

dimnames=list(rownames(exp),colnames(exp))

data=matrix(as.numeric(as.matrix(exp)),nrow=nrow(exp),dimnames=dimnames)

data=avereps(data)

data=data[rowMeans(data)>0.5,]

group=sapply(strsplit(colnames(data),"\\-"), "[", 4)

group=sapply(strsplit(group,""), "[", 1)

group=gsub("2", "1", group)

data=data[,group==0]

data=t(data)

rownames(data)=gsub("(.*?)\\-(.*?)\\-(.*?)\\-.*", "\\1\\-\\2\\-\\3", rownames(data))

data=t(avereps(data))

Risk=read.table(riskFile, header=T, sep="\t", check.names=F, row.names=1)

data=data[,row.names(Risk)]

dataL=data[,row.names(Risk[Risk[,"Risk"]=="low",])]

dataH=data[,row.names(Risk[Risk[,"Risk"]=="high",])]

meanL=rowMeans(dataL)

meanH=rowMeans(dataH)

meanL[meanL<0.00001]=0.00001

meanH[meanH<0.00001]=0.00001

logFC=log2(meanH)-log2(meanL)

logFC=sort(logFC,decreasing=T)

genes=names(logFC)

gmt=read.gmt(gmtFile)

kk=GSEA(logFC, TERM2GENE=gmt, pvalueCutoff = 1)

kkTab=as.data.frame(kk)

kkTab=kkTab[kkTab$pvalue<0.05,]

write.table(kkTab,file="GSEA.result.txt",sep="\t",quote=F,row.names = F)

termNum=5

kkUp=kkTab[kkTab$NES>0,]

if(nrow(kkUp)>=termNum){

showTerm=row.names(kkUp)[1:termNum]

gseaplot=gseaplot2(kk, showTerm, base_size=8, title="Enriched in high risk group")

pdf(file="GSEA.highRisk.pdf", width=7, height=5.5)

print(gseaplot)

dev.off()

}

termNum=5

kkDown=kkTab[kkTab$NES<0,]

if(nrow(kkDown)>=termNum){

showTerm=row.names(kkDown)[1:termNum]

gseaplot=gseaplot2(kk, showTerm, base_size=8, title="Enriched in low risk group")

pdf(file="GSEA.lowRisk.pdf", width=7, height=5.5)

print(gseaplot)

dev.off()

}

library(limma)

library(GSEABase)

library(GSVA)

library(pheatmap)

expFile="symbol.txt"

riskFile="risk.TCGA.txt"

gmtFile="c2.cp.kegg.v7.4.symbols.gmt"

setwd("G:\\")

rt=read.table(expFile, header=T, sep="\t", check.names=F)

rt=as.matrix(rt)

rownames(rt)=rt[,1]

exp=rt[,2:ncol(rt)]

dimnames=list(rownames(exp), colnames(exp))

data=matrix(as.numeric(as.matrix(exp)), nrow=nrow(exp), dimnames=dimnames)

data=avereps(data)

geneSets=getGmt(gmtFile, geneIdType=SymbolIdentifier())

gsvaResult=gsva(data,

geneSets,

min.sz=10,

max.sz=500,

verbose=TRUE,

parallel.sz=1)

data=t(gsvaResult)

group=sapply(strsplit(row.names(data),"\\-"), "[", 4)

group=sapply(strsplit(group,""), "[", 1)

group=gsub("2", "1", group)

data=data[group==0,]

row.names(data)=gsub("(.*?)\\-(.*?)\\-(.*?)\\-(.*?)\\-.*", "\\1\\-\\2\\-\\3", row.names(data))

data=avereps(data)

risk=read.table(riskFile, header=T, sep="\t", check.names=F, row.names=1)

sameSample=intersect(row.names(data), row.names(risk))

data=data[sameSample,,drop=F]

risk=risk[sameSample,"Risk",drop=F]

gsvarisk=cbind(data, risk)

con=gsvarisk[gsvarisk$Risk=="low",]

treat=gsvarisk[gsvarisk$Risk=="high",]

data=rbind(con, treat)

Type=as.vector(data$Risk)

Type=factor(Type, levels=c("low", "high"))

ann=data[,ncol(data),drop=F]

data=t(data[,-ncol(data),drop=F])

design=model.matrix(~0+factor(Type))

colnames(design)=levels(factor(Type))

fit=lmFit(data, design)

cont.matrix=makeContrasts(high-low, levels=design)

fit2=contrasts.fit(fit, cont.matrix)

fit2=eBayes(fit2)

allDiff=topTable(fit2,adjust='fdr',number=200000)

allDiffOut=rbind(id=colnames(allDiff),allDiff)

write.table(allDiffOut, file="all.txt", sep="\t", quote=F, col.names=F)

diffSig=allDiff[with(allDiff, (abs(logFC)>0.1 & adj.P.Val < 0.05)), ]

diffSigOut=rbind(id=colnames(diffSig), diffSig)

write.table(diffSigOut, file="diff.txt", sep="\t", quote=F, col.names=F)

ann_colors=list()

bioCol=c("blue", "red")

names(bioCol)=c("low", "high")

ann_colors[["Risk"]]=bioCol

termNum=50

diffTermName=as.vector(rownames(diffSig))

diffLength=length(diffTermName)

if(diffLength<termNum){termNum=diffLength}

hmGene=diffTermName[1:termNum]

hmExp=data[hmGene,]

pdf(file="heatmap.pdf", width=10, height=6)

pheatmap(hmExp,

annotation=ann,

annotation_colors = ann_colors,

color = colorRampPalette(c(rep("blue",3), "white", rep("red",3)))(50),

cluster_cols=F,

show_colnames = F,

gaps_col=as.vector(cumsum(table(Type))),

scale="row",

fontsize = 7,

fontsize_row=6,

fontsize_col=7)

dev.off()

library(limma)

library(pheatmap)

riskFile="risk.txt"

immFile="infiltration_estimation_for_tcga.csv"

setwd("G:\\")

risk=read.table(riskFile, header=T, sep="\t", check.names=F, row.names=1)

risk$riskScore[risk$riskScore>quantile(risk$riskScore,0.99)]=quantile(risk$riskScore,0.99)

immune=read.csv(immFile, header=T, sep=",", check.names=F, row.names=1)

immune=as.matrix(immune)

rownames(immune)=gsub("(.*?)\\-(.*?)\\-(.*?)\\-(.*)", "\\1\\-\\2\\-\\3", rownames(immune))

immune=avereps(immune)

sameSample=intersect(row.names(risk), row.names(immune))

risk=risk[sameSample, c("risk", "riskScore")]

immune=immune[sameSample,]

data=cbind(risk, immune)

outTab=data.frame()

sigCell=c("risk","riskScore")

for(i in colnames(data)[3:ncol(data)]){

if(sd(data[,i])<0.001){next}

wilcoxTest=wilcox.test(data[,i] ~ data[,"risk"])

pvalue=wilcoxTest$p.value

if(wilcoxTest$p.value<0.05){

outTab=rbind(outTab,cbind(immune=i, pvalue))

sigCell=c(sigCell, i)

}

}

write.table(file="immuneCor.txt", outTab, sep="\t", quote=F, row.names=F)

data=data[,sigCell]

data=data[order(data[,"riskScore"]),]

annCol=data[,1:2]

annCol[,"risk"]=factor(annCol[,"risk"], unique(annCol[,"risk"]))

data=t(data[,(3:ncol(data))])

annRow=sapply(strsplit(rownames(data),"_"), '[', 2)

annRow=as.data.frame(annRow)

row.names(annRow)=row.names(data)

colnames(annRow)=c("Methods")

annRow[,"Methods"]=factor(annRow[,"Methods"], unique(annRow[,"Methods"]))

gapCol=as.vector(cumsum(table(annCol[,"risk"])))

gapRow=as.vector(cumsum(table(annRow[,"Methods"])))

risk=c("blue", "red")

names(risk)=c("low", "high")

ann_colors=list(risk=risk)

pdf("immHeatmap.pdf", width=9, height=6)

pheatmap(data,

annotation=annCol,

annotation_row=annRow,

annotation_colors = ann_colors,

color = colorRampPalette(c(rep("blue",5), "white", rep("red",5)))(100),

cluster_cols =F,

cluster_rows =F,

gaps_row=gapRow,

gaps_col=gapCol,

scale="row",

show_colnames=F,

show_rownames=T,

fontsize=6,

fontsize_row=5,

fontsize_col=6)

dev.off()

library(limma)

library(reshape2)

library(ggpubr)

setwd("G:\\")

scoreCor=function(riskFile=null, scoreFile=null, project=null){

data=read.table(scoreFile, header=T, sep="\t", check.names=F, row.names=1)

data=t(data)

risk=read.table(riskFile, header=T, sep="\t", check.names=F, row.names=1)

sameSample=intersect(row.names(data),row.names(risk))

data=data[sameSample,,drop=F]

risk=risk[sameSample,,drop=F]

rt=cbind(data,risk[,c("riskScore","risk")])

rt=rt[,-(ncol(rt)-1)]

immCell=c("aDCs","B_cells","CD8+_T_cells","DCs","iDCs","Macrophages",

"Mast_cells","Neutrophils","NK_cells","pDCs","T_helper_cells",

"Tfh","Th1_cells","Th2_cells","TIL","Treg")

rt1=rt[,c(immCell,"risk")]

data=melt(rt1,id.vars=c("risk"))

colnames(data)=c("Risk","Type","Score")

data$Risk=factor(data$Risk, levels=c("low","high"))

p=ggboxplot(data, x="Type", y="Score", color = "Risk",

xlab="",ylab="Score",add = "none",palette = c("blue","red") )

p=p+rotate_x_text(50)

p=p+stat_compare_means(aes(group=Risk),symnum.args=list(cutpoints = c(0, 0.001, 0.01, 0.05, 1), symbols = c("***", "**", "*", "")),label = "p.signif")

pdf(file=paste0(project,".immCell.pdf"), width=7, height=6)

print(p)

dev.off()

immFunction=c("APC_co_inhibition","APC_co_stimulation","CCR",

"Check-point","Cytolytic_activity","HLA","Inflammation-promoting",

"MHC_class_I","Parainflammation","T_cell_co-inhibition",

"T_cell_co-stimulation","Type_I_IFN_Reponse","Type_II_IFN_Reponse")

rt1=rt[,c(immFunction,"risk")]

data=melt(rt1,id.vars=c("risk"))

colnames(data)=c("Risk","Type","Score")

data$Risk=factor(data$Risk, levels=c("low","high"))

p=ggboxplot(data, x="Type", y="Score", color = "Risk",

xlab="",ylab="Score",add = "none",palette = c("blue","red") )

p=p+rotate_x_text(50)

p=p+stat_compare_means(aes(group=Risk),symnum.args=list(cutpoints = c(0, 0.001, 0.01, 0.05, 1), symbols = c("***", "**", "*", "")),label = "p.signif")

pdf(file=paste0(project,".immFunction.pdf"), width=7, height=6)

print(p)

dev.off()

}

scoreCor(riskFile="trainRisk.txt", scoreFile="TCGA.score.txt", project="TCGA")

scoreCor(riskFile="testRisk.txt", scoreFile="GEO.score.txt", project="GEO")

library(survival)

library(survminer)

setwd("G:\\")

risk=read.table("risk.txt",header=T,sep="\t",check.names=F,row.names=1)

cli=read.table("clinical.txt",sep="\t",check.names=F,header=T,row.names=1)

sameSample=intersect(row.names(cli),row.names(risk))

risk=risk[sameSample,]

cli=cli[sameSample,]

data=cbind(futime=risk[,1],fustat=risk[,2],cli,risk=risk[,"risk"])

for(i in colnames(data[,3:(ncol(data)-1)])){

rt=data[,c("futime","fustat",i,"risk")]

rt=rt[(rt[,i]!="unknow"),]

colnames(rt)=c("futime","fustat","clinical","risk")

tab=table(rt[,"clinical"])

tab=tab[tab!=0]

for(j in names(tab)){

rt1=rt[(rt[,"clinical"]==j),]

tab1=table(rt1[,"risk"])

tab1=tab1[tab1!=0]

labels=paste0(names(tab1)," risk(n=",tab1,")")

if(length(labels)==2){

titleName=j

if((i=="age") | (i=="Age") | (i=="AGE")){

titleName=paste0("age",j)

}

diff=survdiff(Surv(futime, fustat) ~risk,data = rt1)

pValue=1-pchisq(diff$chisq,df=1)

if(pValue<0.001){

pValue="p<0.001"

}else{

pValue=paste0("p=",sprintf("%.03f",pValue))

}

fit <- survfit(Surv(futime, fustat) ~ risk, data = rt1)

surPlot=ggsurvplot(fit,

data=rt1,

pval=pValue,

pval.size=6,

legend.labs=labels,

legend.title=titleName,

font.legend=13,

xlab="Time(years)",

break.time.by = 1,

palette=c("red","blue") )

j=gsub(">=","ge",j);j=gsub("<=","le",j);j=gsub(">","gt",j);j=gsub("<","lt",j)

pdf(file=paste0("survival.",i,"_",j,".pdf"),onefile = FALSE,

width = 5,

height =4.5)

print(surPlot)

dev.off()

}

}

}

library(limma)

library(ggpubr)

library(pRRophetic)

library(ggplot2)

set.seed(12345)

pFilter=0.001

expFile="symbol.txt"

riskFile="risk.TCGA.txt"

setwd("G:\\A")

data(cgp2016ExprRma)

data(PANCANCER_IC_Tue_Aug_9_15_28_57_2016)

allDrugs=unique(drugData2016$Drug.name)

rt = read.table(expFile, header=T, sep="\t", check.names=F)

rt=as.matrix(rt)

rownames(rt)=rt[,1]

exp=rt[,2:ncol(rt)]

dimnames=list(rownames(exp),colnames(exp))

data=matrix(as.numeric(as.matrix(exp)),nrow=nrow(exp),dimnames=dimnames)

data=avereps(data)

data=data[rowMeans(data)>0.5,]

group=sapply(strsplit(colnames(data),"\\-"), "[", 4)

group=sapply(strsplit(group,""), "[", 1)

group=gsub("2","1",group)

data=data[,group==0]

data=t(data)

rownames(data)=gsub("(.*?)\\-(.*?)\\-(.*?)\\-(.*)", "\\1\\-\\2\\-\\3", rownames(data))

data=avereps(data)

data=t(data)

riskRT=read.table(riskFile, header=T, sep="\t", check.names=F, row.names=1)

riskRT$riskScore[riskRT$riskScore>quantile(riskRT$riskScore,0.99)]=quantile(riskRT$riskScore,0.99)

for(drug in allDrugs){

possibleError=tryCatch(

{senstivity=pRRopheticPredict(data, drug, selection=1, dataset = "cgp2016")},

error=function(e) e)

if(inherits(possibleError, "error")){next}

senstivity=senstivity[senstivity!="NaN"]

senstivity[senstivity>quantile(senstivity,0.99)]=quantile(senstivity,0.99)

sameSample=intersect(row.names(riskRT), names(senstivity))

risk=riskRT[sameSample, c("riskScore","Risk"),drop=F]

senstivity=senstivity[sameSample]

rt=cbind(risk, senstivity)

rt$Risk=factor(rt$Risk, levels=c("low", "high"))

type=levels(factor(rt[,"Risk"]))

comp=combn(type, 2)

my_comparisons=list()

for(i in 1:ncol(comp)){my_comparisons[[i]]<-comp[,i]}

test=wilcox.test(senstivity~Risk, data=rt)

diffPvalue=test$p.value

x=as.numeric(rt[,"riskScore"])

y=as.numeric(rt[,"senstivity"])

corT=cor.test(x, y, method="spearman")

corPvalue=corT$p.value

if((diffPvalue<pFilter) & (corPvalue<pFilter)){

boxplot=ggboxplot(rt, x="Risk", y="senstivity", fill="Risk",

xlab="Risk",

ylab=paste0(drug, " senstivity (IC50)"),

legend.title="Risk",

palette=c("#0066FF","#FF0000")

)+

stat_compare_means(comparisons=my_comparisons)

pdf(file=paste0("durgSenstivity.", drug, ".pdf"), width=5, height=4.5)

print(boxplot)

dev.off()

df1=as.data.frame(cbind(x,y))

p1=ggplot(df1, aes(x, y)) +

xlab("Risk score") + ylab(paste0(drug, " senstivity (IC50)"))+

geom_point() + geom_smooth(method="lm",formula = y ~ x) + theme_bw()+

stat_cor(method = 'spearman', aes(x =x, y =y))

pdf(file=paste0("Cor.", drug, ".pdf"), width=5, height=4.6)

print(p1)

dev.off()

}

}

library(limma)

library(ggpubr)

riskFile="risk.IMvigor.txt"

cliFile="clinical.txt"

setwd("G:\\")

risk=read.table(riskFile, header=T, sep="\t", check.names=F, row.names=1)

risk$riskScore[risk$riskScore>quantile(risk$riskScore,0.99)]=quantile(risk$riskScore,0.99)

cli=read.table(cliFile, header=T, sep="\t", check.names=F, row.names=1)

samSample=intersect(row.names(risk), row.names(cli))

risk=risk[samSample,"riskScore",drop=F]

cli=cli[samSample,,drop=F]

rt=cbind(risk, cli)

for(clinical in colnames(rt)[2:ncol(rt)]){

data=rt[c("riskScore", clinical)]

colnames(data)=c("riskScore", "clinical")

data=data[(data[,"clinical"]!="unknow"),]

group=levels(factor(data$clinical))

data$clinical=factor(data$clinical, levels=group)

comp=combn(group,2)

my_comparisons=list()

for(i in 1:ncol(comp)){my_comparisons[[i]]<-comp[,i]}

boxplot=ggboxplot(data, x="clinical", y="riskScore", color="clinical",

xlab="",

ylab="Risk score",

legend.title=clinical,

add = "jitter")+

stat_compare_means(comparisons = my_comparisons)

#stat_compare_means(comparisons = my_comparisons,symnum.args=list(cutpoints = c(0, 0.001, 0.01, 0.05, 1), symbols = c("***", "**", "*", "ns")),label = "p.signif")

pdf(file=paste0("cliCor.", clinical, ".pdf"), width=6, height=5)

print(boxplot)

dev.off()

}
