# Supplementary material for: Identification and validation of the nicotine metabolism-related signature of bladder cancer by bioinformatics and machine learning
Source: Front Immunol. 2024 Dec 17;15:1465638. doi: 10.3389/fimmu.2024.1465638 (PMC11685211; doi:10.3389/fimmu.2024.1465638)
Supplement: Supplementary file 1 [file DataSheet1.zip › Supplementary Material.docx]

***Supplementary Material***

***Identification and validation of the nicotine metabolism-related signature of bladder cancer by bioinformatics and machine learning***

Yating Zhan^1, a^, Min Weng^2, a^, Yangyang Guo^3, a^, Dingfeng Lv^1^, Feng Zhao^1^, Zejun Yan^2^, Junhui Jiang^2, *^, Yanyi Xiao^4, *^, Lili Yao^5, *^

***Correspondence:** Lili Yao, yaolili9813@126.com; Yanyi Xiao, 15967411128@163.com; Junhui Jiang, jiangjh200509@126.com.

**1 Supplementary Figures and Tables**

**1.1 Supplementary Figures**


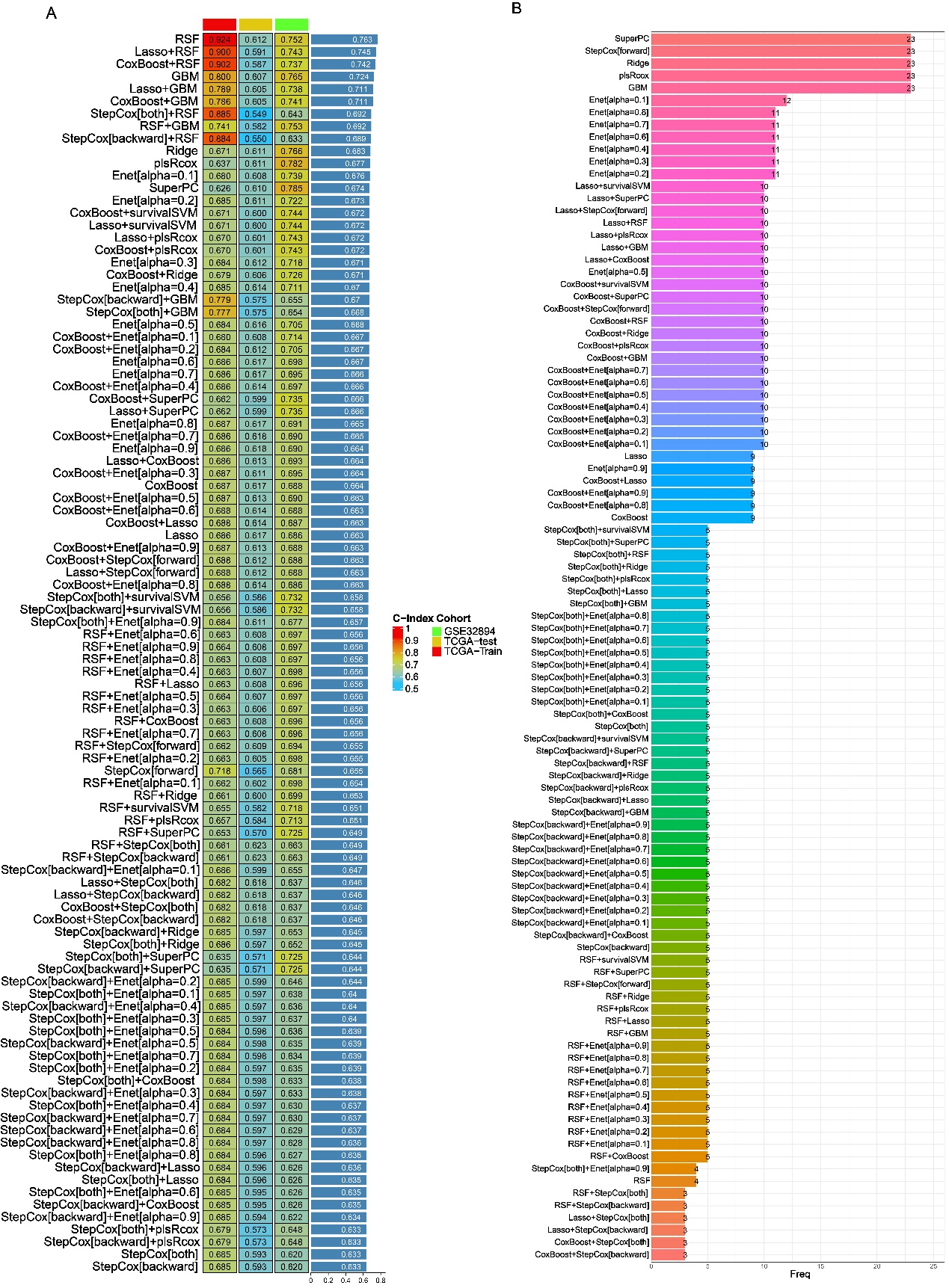


**Supplementary Figure S1** Construction of NRS with integrative machine learning algorithms. (A) The C-index of 100 kinds of prognostic signatures constructed with 10 machine learning algorithms in TCGA-train, TCGA-test and GSE32894 cohorts. (B) The gene frequency of 100 kinds of prognostic signatures.


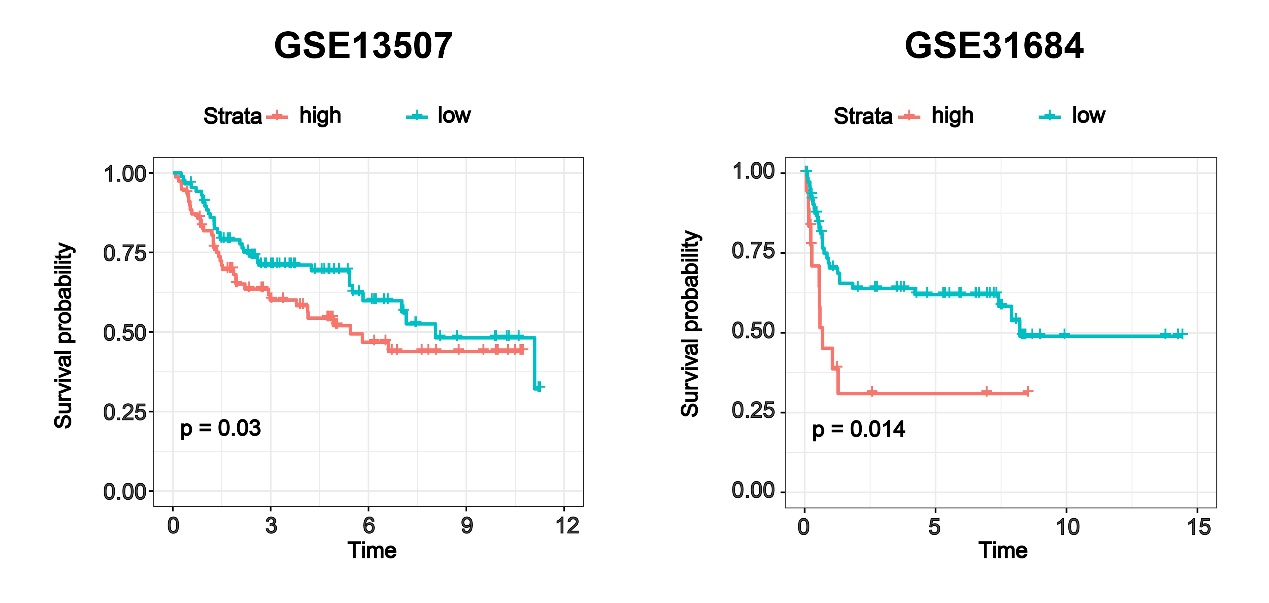


**Supplementary Figure S2** K-M survival curves of BLCA patients across GSE13507 and GSE31684 datasets.


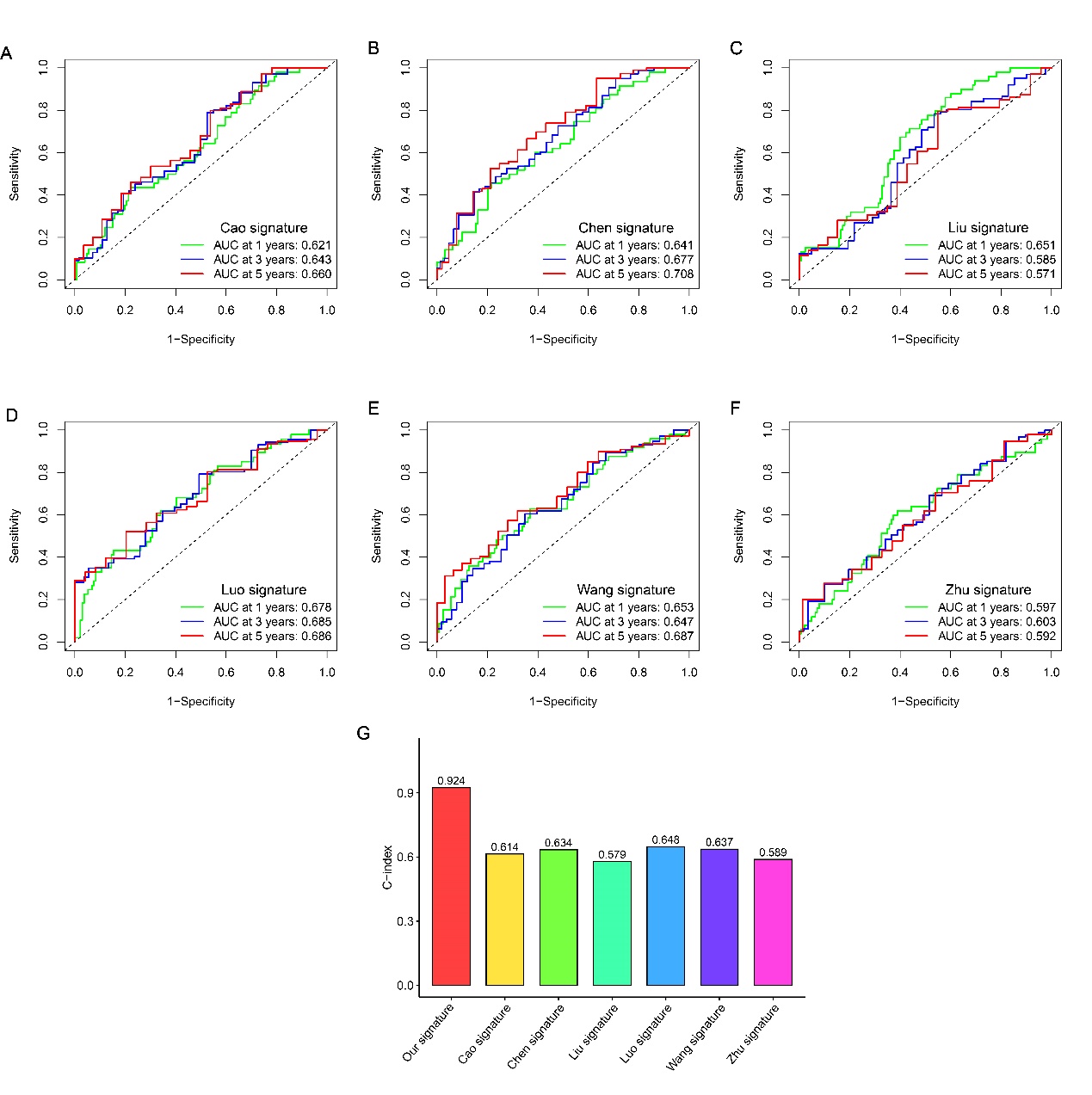


**Supplementary Figure S3** Comparisons of the predictive value of NRS with other 6 published signatures. (A-F) The ROC curves of other 6 published signatures. (G) The C-index comparing the predictive value of NRS and other 6 published signatures.


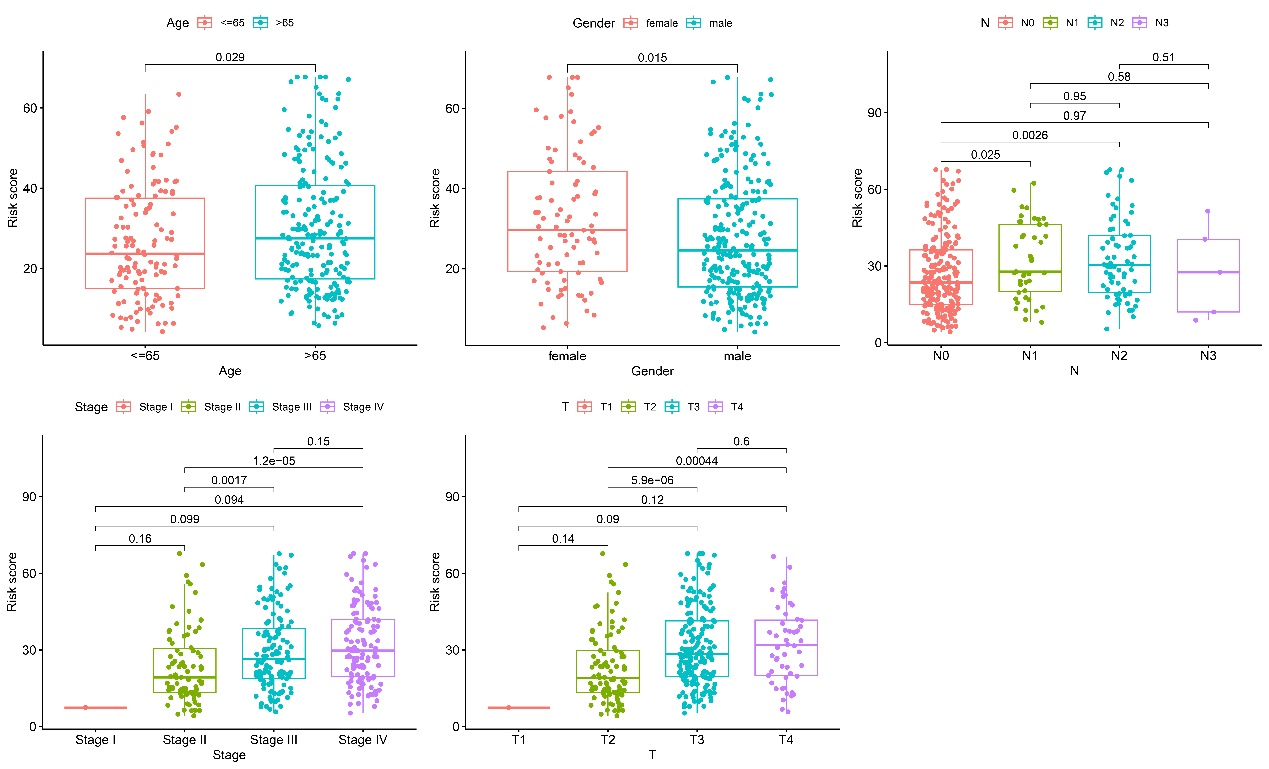


**Supplementary Figure S4** Correlation analysis of NRS with clinical characteristics (age, gender, N stage, tumor stage, and T stage).


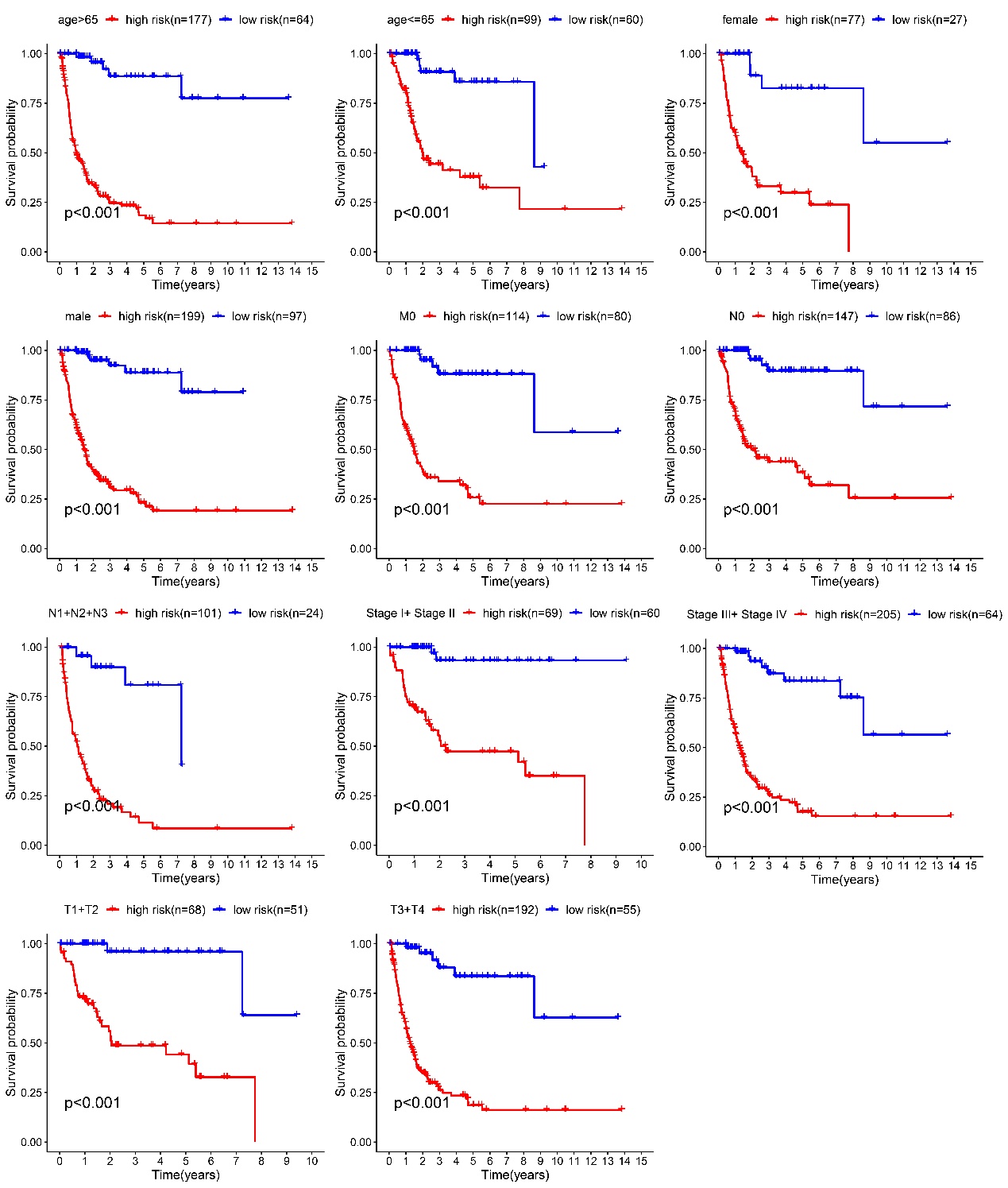


**Supplementary Figure S5** Stratified survival analysis between high- and low-NRS patients in subgroups of age > 65 years, age ≤65 years, female, male, M0 stage, N0 stage, N1-3 stage, tumor stage I–II, tumor stage III–IV, T1-2 stage and T3-4 stage.

**1.2 Supplementary Tables**

**Supplementary Table S1** The gene list of NRS.

**1.3 Supplementary Methods**

**Machine learning algorithms developed a prognostic NRS**

10 machine learning algorithms (stepwise Cox, random survival forest [RSF], elastic network [Enet], supervised principal components [SuperPC], partial least squares regression for Cox [plsRcox], CoxBoost, survival support vector machine [survival-SVM], Lasso, Ridge and generalized boosted regression modeling [GBM]) were integrated into 100 kinds of machine learning combination. The stepwise Cox model was preformed via survival package. Stepwise Cox regression is a stepwise variable selection process based on the Akaike Information Criterion (AIC), identifying variables that significantly influence survival time to construct an optimal Cox proportional hazards model. RSF was preformed via randomForestSRC package. RSF included two parameters, ntree and mtry. The ntree was the number of trees in the forest and mtry represented the number of variables randomly selected for splitting at each node. A grid search was conducted for ntree and mtry using 10-fold cross-validation. All pairs of (ntree, mtry) were obtained, and the pair with the highest C-index value was determined as the optimization parameter. Enet, Lasso and Ridge were preformed via glmnet package. All the three algorithms contribute to addressing the problem of overfitting. SuperPC was implemented via superpc package. SuperPC is an improvement of principal component analysis (PCA), focusing on dimension reduction of variables that are closely associated with survival time. The plsRcox model was implemented via plsRcox package. The plsRcox extracts the most relevant features and constructs a generalized linear model using partial least squares regression. CoxBoost was preformed via CoxBoost package. CoxBoost is a Cox regression model based on boosting algorithms, fitting the Cox proportional hazards model through componentwise likelihood-based boosting. Survival-SVM was preformed via survivalsvm package. Survival-SVM is an extension of SVM for survival data analysis, predicting survival time and risk levels by maximizing the decision boundary of the data. The GBM model was implemented via the gbm package. GBM is an ensemble learning method that iteratively combines multiple weak learners, typically decision trees, to enhance the accuracy and robustness of model.
